# Impact of alemtuzumab-mediated lymphocyte depletion on SIV reservoir establishment and persistence

**DOI:** 10.1371/journal.ppat.1012496

**Published:** 2024-08-22

**Authors:** Benjamin Varco-Merth, Morgan Chaunzwa, Derick M. Duell, Alejandra Marenco, William Goodwin, Rachel Dannay, Michael Nekorchuk, Danica Shao, Kathleen Busman-Sahay, Christine M. Fennessey, Lorna Silipino, Michael Hull, William J. Bosche, Randy Fast, Kelli Oswald, Rebecca Shoemaker, Rachele Bochart, Rhonda MacAllister, Caralyn S. Labriola, Jeremy V. Smedley, Michael K. Axthelm, Miles P. Davenport, Paul T. Edlefsen, Jacob D. Estes, Brandon F. Keele, Jeffrey D. Lifson, Sharon R. Lewin, Louis J. Picker, Afam A. Okoye

**Affiliations:** 1 Vaccine and Gene Therapy Institute, Oregon Health & Science University, Beaverton, Oregon, United States of America; 2 Oregon National Primate Research Center, Oregon Health & Science University, Beaverton, Oregon, United States of America; 3 Fred Hutchinson Cancer Research Center, Seattle, Washington State, United States of America; 4 AIDS and Cancer Virus Program, Frederick National Laboratory for Cancer Research, Frederick, Maryland, United States of America; 5 Kirby Institute, University of New South Wales, Sydney, New South Wales, Australia; 6 Department of Infectious Diseases, The University of Melbourne at The Peter Doherty Institute for Infection and Immunity, Melbourne, Australia; 7 Victorian Infectious Diseases Service, Royal Melbourne Hospital at The Peter Doherty Institute for Infection and Immunity, Melbourne, Australia; 8 Department of Infectious Diseases, Alfred Hospital and Monash University, Melbourne, Australia; University of Wisconsin, UNITED STATES OF AMERICA

## Abstract

Persistence of the rebound-competent viral reservoir (RCVR) within the CD4^+^ T cell compartment of people living with HIV remains a major barrier to HIV cure. Here, we determined the effects of the pan-lymphocyte-depleting monoclonal antibody (mAb) alemtuzumab on the RCVR in SIVmac239-infected rhesus macaques (RM) receiving antiretroviral therapy (ART). Alemtuzumab administered during chronic ART or at the time of ART initiation induced >95% depletion of circulating CD4^+^ T cells in peripheral blood and substantial CD4^+^ T cell depletion in lymph nodes. However, treatment was followed by proliferation and reconstitution of CD4^+^ T cells in blood, and despite ongoing ART, levels of cell-associated SIV DNA in blood and lymphoid tissues were not substantially different between alemtuzumab-treated and control RM after immune cell reconstitution, irrespective of the time of alemtuzumab treatment. Upon ART cessation, 19 of 22 alemtuzumab-treated RM and 13 of 13 controls rebounded with no difference in the time to rebound between treatment groups. Time to rebound and reactivation rate was associated with plasma viral loads (pVLs) at time of ART initiation, suggesting lymphocyte depletion had no durable impact on the RCVR. However, 3 alemtuzumab-treated RM that had lowest levels of pre-ART viremia, failed to rebound after ART withdrawal, in contrast to controls with similar levels of SIV replication. These observations suggest that alemtuzumab therapy has little to no ability to reduce well-established RCVRs but may facilitate RCVR destabilization when pre-ART virus levels are particularly low.

## Author summary

A major challenge to curing HIV is the presence of cells containing the rebound-competent viral reservoirs (RCVR) capable of re-establishing systemic infection when ART is interrupted. CD4^+^ T cells harboring RCVR persist for life due to homeostatic mechanisms that allow them to proliferate and expand during ART. In this study, we explored whether pan-lymphocyte depletion with a licensed CD52-depleting monoclonal antibody called alemtuzumab, can eliminate or reduce RCVR in SIV-infected rhesus macaques (RM) when administered either at the time of ART initiation or during chronic ART administration. While alemtuzumab induced massive depletion of lymphocytes in blood, including >95% depletion of CD4^+^ T cells, there were no measurable effects of alemtuzumab on cell-associated viral loads during ART or SIV rebound dynamics following ART interruption in most RM. Three alemtuzumab-treated RM failed to manifest rebound viremia during extended follow up after ART withdrawal. However, these animals had the lowest levels of pre-ART SIV replication, suggesting that alemtuzumab may have been effective in RM with early and presumably smaller RCVR. Overall, our study highlights the current limitations of lymphocyte depletion strategies in facilitating the permanent disruption of RCVR, particularly once the RCVR is established above levels that induce consistent post-ART viremia.

## Introduction

HIV remains a global public health concern, as an estimated 39 million people are currently living with HIV worldwide and 1.3 million were newly infected in 2022 [1]. Although antiretroviral therapy (ART) has significantly improved survival and the quality of life for people with HIV (PWH), and “treatment as prevention” has helped to reduce viral transmission, ART alone is not a cure and requires lifelong adherence to prevent new cycles of virus replication and resumption of disease progression. Failure to cure HIV infection is due, in large part, to a population of predominantly memory CD4^+^ T cells (T_M_) harboring integrated, replication-competent HIV proviruses. This reservoir of “latently-infected” cells can persist for long periods of time and be maintained by homeostatic mechanisms [2–4]. As such, the elimination of latently-infected cells in PWH is a priority for ongoing efforts to establish more definitive treatments that would obviate the need for ongoing ART, or even a cure for HIV infection. However, identifying cells harboring the rebound-competent viral reservoir (RCVR) in order to target them for elimination has been a major challenge. One approach is to induce widespread lymphocyte depletion, which could potentially include depletion of the CD4^+^ T cell-based RCVR, reducing its size while ART protects from new rounds of cell infection. Indeed, durable remission from HIV replication following ART interruption (ATI) has been reported in PWH that received myeloablative conditioning prior to allogeneic stem cell transplantation. However, most of these documented cures were achieved in the setting of donors with the CCR5-delta32 genotype [5–7]. This suggests that the combination of immune ablation and subsequent donor cell chimerism prevented viral rebound after ATI due to a paucity of viral target cells (CD4^+^ CCR5^+^ T cells). An extended period of HIV remission following ATI was also reported in two subjects who received myeloablative conditioning and transplantation of allogeneic stem cell without the HIV-resistant CCR5-delta32 genotype [8]. Although viral rebound eventually occurred several weeks following ATI, these cases suggest that lymphocyte depletion in the presence of ART, may reduce RCVR levels sufficiently to delay rebound, but also cautions that even a greatly reduced RCVR may eventually enable viral recrudescence.

Allogeneic stem cell transplantation is clearly impractical as a global strategy to cure HIV infection, but oral or intravenous agents capable of targeting cells harbouring the RCVR could be of potential interest. Indeed, targeted depletion of specific lymphocyte populations with monoclonal antibodies (mAb) is extensively used to treat several disease conditions. Rituximab, a mAb that targets CD20^+^ B cells is approved for the treatment of autoimmune disorders such as rheumatoid arthritis and hematological malignancies, including non-Hodgkin’s lymphoma and chronic lymphocytic leukemia [9–12]. Similarly, anti-thymocyte globulins are lymphocyte-depleting polyclonal antibodies used in the treatment of renal allograft rejection [13–15]. Modifications to depleting mAb have been employed to improve efficiency and reduce off-target effects. For example, Brentuximab vedotin is a drug-antibody conjugate that targets cells expressing CD30 and is currently used to treat both Hodgkin’s and non-Hodgkin’s lymphoma [16–19]. However, while lymphocyte depleting strategies have potential for RCVR elimination in PWH, there are challenges with their adoption for HIV cure, including: 1) the absence of a cellular marker that is specifically enriched on cells with RCVR [20]. This limits the development of mAbs designed to specifically target latently-infected cells, 2) off-target lymphocyte depletion would impact populations required for antiviral immune surveillance, such as CD8^+^ T cells and NK cells, 3) homeostatic proliferation, which is an important mechanism of HIV persistence during therapy, could also promote the reconstitution and/or further expansion of cells harboring RCVR, and 4) reservoir depletion strategies may have limited efficacy if they are ineffective at targeting RCVR in tissues.

Despite these issues, the question remains as to whether a lymphocyte depletion strategy can not only reduce the size and distribution of RCVR virus in PWH who initiate ART during chronic HIV infection, with presumptive “late/large reservoirs”, but might have potential to facilitate complete RCVR eradication in PWH who initiate ART during acute infection, with presumptive “early/low reservoirs”. To evaluate this, we used alemtuzumab, a humanized lymphocyte-depleting mAb that targets CD52^+^ cells [21–23]. Alemtuzumab (previously known as CAMPATH-1H) was approved in 2001 to treat chronic lymphocytic leukemia [22,24,25]. It is currently marketed as Lemtrada and is approved for the treatment of relapsing forms of multiple sclerosis [26]. CD52, also known as CAMPATH-1 antigen, is a 12-amino acid glycosylphosphatidylinositol-linked glycoprotein [27]. In humans, CD52 is expressed at high levels on B and T lymphocytes and at lower levels on NK cells [28]. While the primary function of CD52 remains poorly defined, it has been shown to bind SIGLEC-10 and suggested to play multiple roles in anti-adhesion and T cell regulation [27,29–31]. Alemtuzumab depletes CD52^+^ cells via antibody dependent cell-mediated cytotoxicity and complement dependent cytotoxicity [32]. A potential role for alemtuzumab against HIV was shown by its ability to target HIV-infected cells isolated from PWH in *ex vivo* assays [33]. Alemtuzumab was also shown to reduce cell-associated viral loads in a person with HIV treated with the drug for Sezary syndrome [34]. Additionally, alemtuzumab was part of the immune conditioning regimen for Adam Castillejo, the London patient, who has been in long-term HIV remission off ART following allogeneic stem cell transplantation [5,35].

In this study, we assessed the impact of alemtuzumab on RCVR dynamics in SIV-infected RM on ART. We demonstrate that alemtuzumab can induce lymphocyte depletion in blood and lymph nodes of RM. However, alemtuzumab treatment during chronic ART or at time of ART initiation had no substantial impact on cell-associated viral loads or SIV rebound dynamics following ATI, except in 3 RM with very low levels of pre-ART viremia. These results, demonstrating limited virologic benefit, despite using a potent, broad lymphocyte depleting reagent capable of depleting not only infected cells, but also uninfected potential target cells in a manner that might be expected to limit viral spread and persistence, underscore the challenges in achieving sufficient depletion of the RCVR to achieve viral remission with such depletion strategies or even more restrictive ones targeting only virus expressing cells.

## Results

### Pilot study

**Fig 1:**
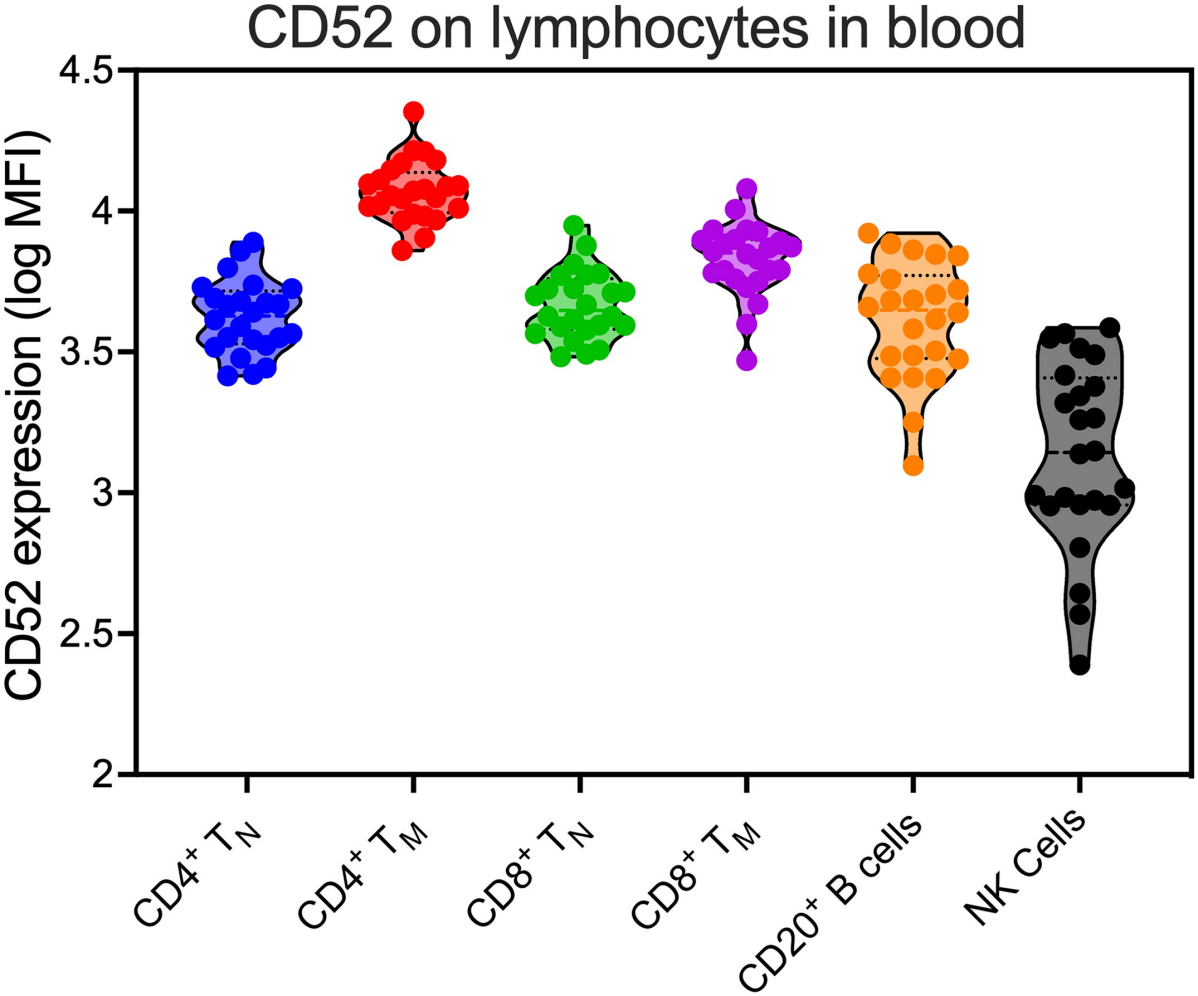
CD52 expression on lymphocytes in blood. CD52 expression as measured by mean fluorescent intensity (MFI) on CD4^+^ naïve (T_N_), CD4^+^ memory (T_M_), CD8^+^ T_N_, CD8^+^ T_M_, CD20^+^ B cells and CD3-NKG2A^+^ NK cells in the blood of SIV-naïve RM (n = 28).

**Fig 2:**
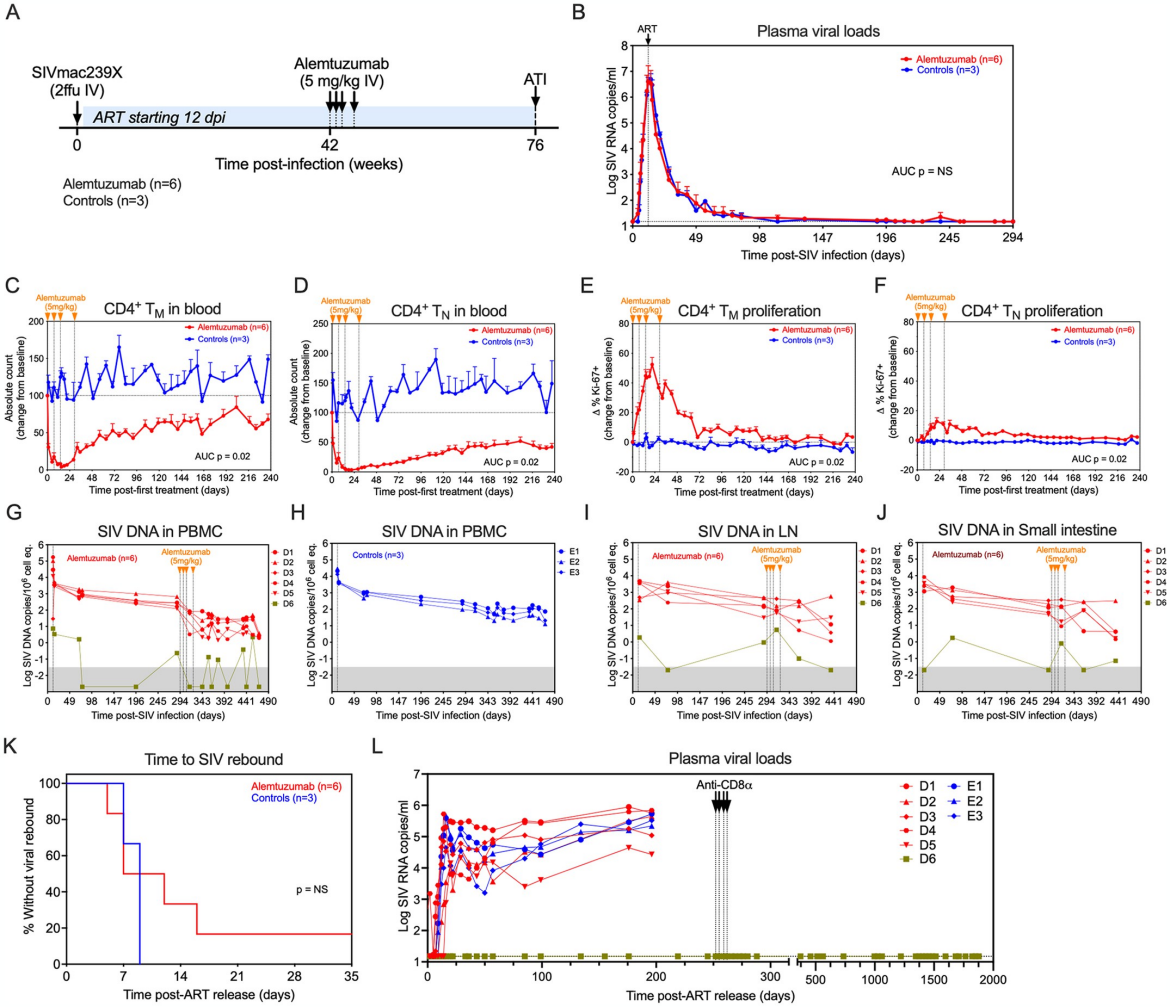
Alemtuzumab depletes circulating CD4^+^ naïve and memory T cells in a pilot study. (A) Schematic representation of the study protocol showing SIVmac239 infection, ART initiation 12 dpi, alemtuzumab treatment at 294-, 301-, 308- and 322-days post-infection (dpi). (B) Mean (+SEM) plasma viral load (pvl) profiles of alemtuzumab-treated RM (n = 6) versus untreated controls (n = 3) prior to antibody infusion. (C—F) Change in absolute counts and change in the proliferative fraction (Ki67+) of CD4+ memory (T_M_) and naïve (T_N_) T cell subsets in blood following alemtuzumab (n = 6) or no treatment (n = 3). Results are shown as mean (+SEM) change from baseline of percentages of baseline absolute counts or percentages of Ki-67. (G and H) Quantification of cell-associated SIV DNA levels in PBMCs (copies per 10^6^ cell equivalents) in alemtuzumab-treated RM and untreated controls. RM D6 with no post-ART rebound viremia is shown in green. (I and J) Quantification of cell-associated SIV DNA levels in peripheral lymph nodes (LN) and small intestine (copies per 10^6^ cell equivalents) in alemtuzumab-treated RM. Threshold sensitivity varied as a function of the number of cells available for analysis; values below threshold are indicated by the gray area. (K) Kaplan-Meier analysis of SIV rebound kinetics in RM treated with alemtuzumab (n = 6) or no treatment (n = 3). (L) Individual pvl profiles of RM in each treatment group following ART release at 530 dpi. The dotted line indicates a pvl threshold of 15 RNA copies/ml. RM D6 received the anti-CD8α depleting mAb MT807R1 at 10, 5, 5, and 5mg/kg on days 252, 255, 259 and 262 post-ART release. The WRS test was used to determine the significance of differences in AUC between the two treatment groups (p-values ≤ 0.05 are shown).

The primary goal of this study was to determine whether lymphocyte depletion with alemtuzumab could reduce or eliminate the RCVR in SIV-infected RM on ART. Alemtuzumab targets CD52, which is expressed on the vast majority of lymphocytes in blood of RM, including CD4^+^ and CD8^+^ T cells, B cells, and NK cells (Fig 1). As CD52 can be expressed on erythrocytes in old-world monkeys [36,37], we performed a pilot study to determine drug safety and to characterize the dynamics of lymphocyte depletion and reconstitution following alemtuzumab treatment during ART-suppressed SIV infection. For this, a total of 9 RM (S1 Table) received an intravenous (IV) inoculation of 2 infectious units (IU) of SIVmac239X followed by ART consisting of tenofovir disoproxil, emtricitabine and dolutegravir, starting 12 days post-infection (dpi) (Fig 2A). RM were then divided into 2 groups that received alemtuzumab at 5mg/kg IV (n = 6) at 42, 43, 44 and 46 weeks post-SIV infection or no treatment (n = 3).

Following SIV-infection, plasma viral loads (pVL) peaked by 14 dpi before declining on ART to below the 15 SIV RNA copies/ml threshold in all RM by 280 dpi, prior to alemtuzumab infusion (Fig 2B). Alemtuzumab treatment resulted in substantial depletion of CD4^+^ T cells in blood, including >95% depletion of CD4^+^ T_N_ and T_M_ subsets (Fig 2C and 2D). Depletion was followed by a marked increase in proliferation of all CD4^+^ T cell subsets (Figs 2E and 2F and S1). A similar pattern of CD8^+^ T cell depletion was observed, which was characterized by a massive loss in CD8^+^ T_N_ and T_M_ in blood followed by an increase in proliferation (S2A–S2D Fig). Alemtuzumab also induced >98% depletion of NK cells and >90% depletion of B cells in blood (S2E and S2F Fig). Collectively, these data demonstrate the potency of alemtuzumab-mediated depletion against circulating CD52^+^ lymphocytes in RM (Fig 1).

Plasma SIV RNA was monitored by a high sensitivity assay from 112 days prior to and during alemtuzumab treatment. Despite the marked increase in CD4^+^ T_M_ proliferation (Fig 2E and 2F), only 1 RM showed an increase in viral blips above 15 SIV RNA copies per ml (standard threshold) during alemtuzumab treatment (S3 Fig). We also assessed the impact of alemtuzumab treatment on levels of persistently-infected cells as measured by cell-associated SIV viral loads. As shown in Fig 2G, levels of SIV DNA in blood of alemtuzumab-treated RM declined slightly post-treatment, which was associated with the transient depletion of CD4^+^ T cells in blood. In contrast, SIV DNA levels in untreated control RM remained stable over time (Fig 2H). Although we did not assess levels of CD4^+^ T cell depletion in lymph node (LN) and small intestine, SIV DNA levels in those tissues were generally stable in most RM following alemtuzumab treatment (Fig 2I and 2J). This may suggest inadequate T cell depletion in those tissues. However, in RM D6 which had the lowest pVL at time of ART (S1 Table), levels of cell-associated SIV DNA in blood and tissues often declined below the threshold of detection before and after alemtuzumab treatment.

After 75 weeks of ART administration, ~34 weeks after alemtuzumab treatment, ART was discontinued to assess the effect of treatment on the dynamics of SIV rebound. Five of 6 alemtuzumab-treated RM manifested SIV rebound in plasma within 16 days of ART release with no difference in the time to rebound between alemtuzumab-treated RM and controls (Fig 2K and 2L). However, RM D6 showed no evidence of post-ART virus rebound. To determine whether the absence of rebound viremia in D6 was associated with CD8^+^ T cell control, we administered the anti-CD8α depleting mAb MT807R1 starting 252 days post-ART release; no increase in pVL was observed following MT807R1 administration (S4 Fig), suggesting that this monkey was not an immunologic viral controller, but rather, had experienced complete loss of its RCVR prior to ART release. Of note, RM D6 had the lowest levels of pre-ART pVL peaking at only 16,000 SIV RNA copies/ml by 14 dpi, within a range previously associated with RCVR instability following low dose IV SIVmac239 challenge and early ART [38]. This suggests that alemtuzumab-mediated depletion may have facilitated permanent disruption of the early RCVR in this RM. While this observation in a single animal is not definitive, this preliminary observation suggested that if the extent of viral seeding prior to ART initiation is constrained below a threshold level where post-ART viral reactivation and rebound are stochastic [38], then lymphocyte depletion may contribute to RCVR depletion below the level necessary for SIV rebound.

### Effects of alemtuzumab on lymphocyte dynamics during chronic ART

**Fig 3:**
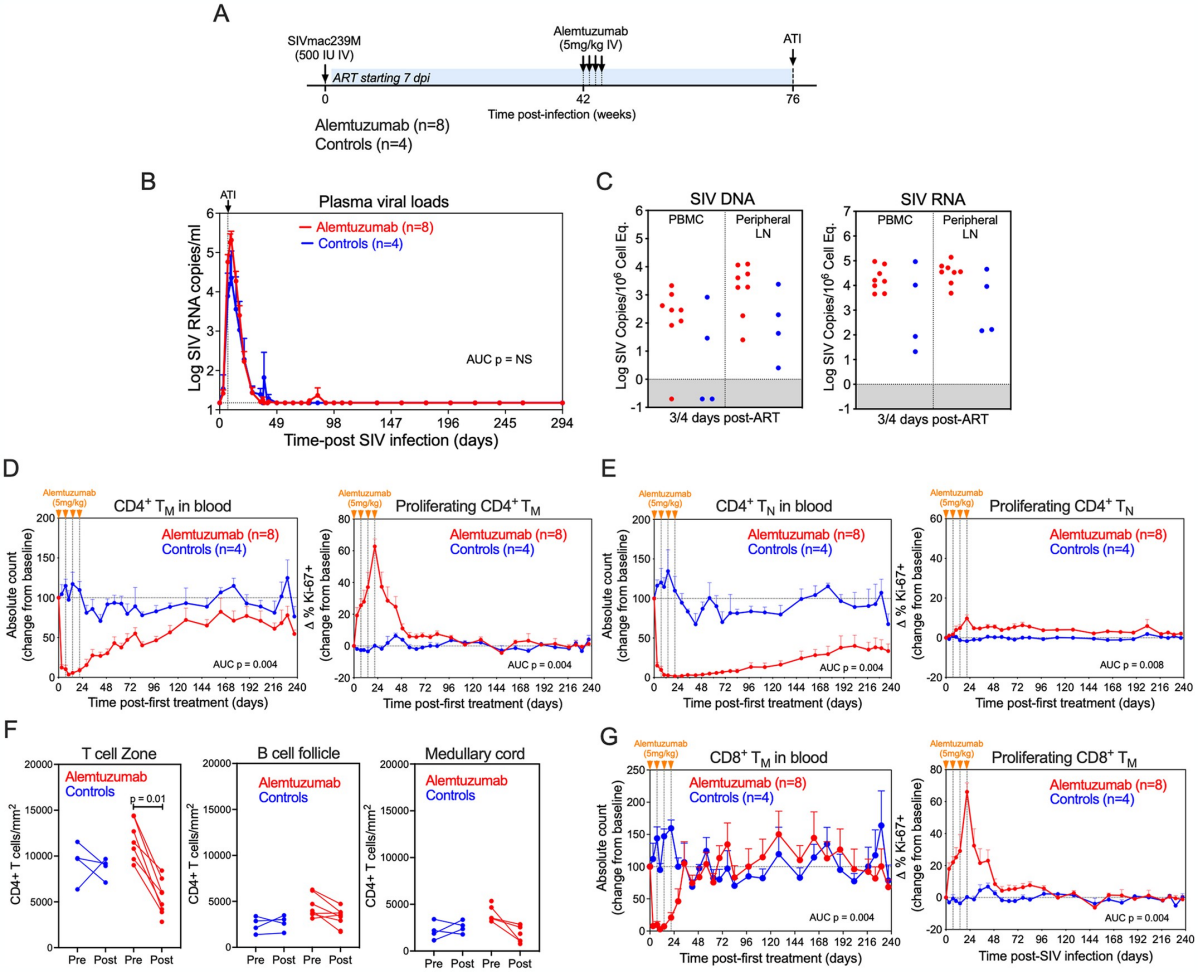
Alemtuzumab depletes CD4^+^ an CD8^+^ T cells in blood and lymph nodes. (A) Schematic representation of the study protocol showing SIVmac239 infection, ART initiation 7 dpi and alemtuzumab (n = 8) or human IgG control mAb (n = 4) treatment at 294, 301, 308, 315 dpi. (B and C) Mean (+SEM) plasma viral load profiles and comparison of SIV DNA (left panel) and SIV RNA (right panel) levels in PBMC and peripheral lymph node (LN) (copies per 10^6^ cell equivalents) 3-4 days after ART initiation of alemtuzumab-treated RM (n = 8) versus human IgG-treated controls (n = 4). Threshold sensitivity varied as a function of the number of cells available for analysis; values below threshold are indicated by the gray area. (D and E) Change in absolute counts (left panel) and change in the proliferative fraction (right panel) of CD4^+^ memory (T_M_) and naïve (T_N_) T cell subsets in blood following alemtuzumab (n = 8) or human IgG control mAb (n = 4). Results are shown as mean (+SEM) change from baseline of percentages of baseline absolute counts or percentages of Ki-67. (F) Quantification of the number of CD4^+^ cells per 1 × 10^5^ cells in T cell zone, B cell follicle and medullary cords of in LN before and after alemtuzumab treatment. LN biopsies were obtained between 7–13 days after the last dose of alemtuzumab. Each data point represents the average number of CD4^+^ cells derived from quantitative measures from 2–3 LN sections from a single time point from an individual RM. (G) Change in absolute counts (left panel) and change in the proliferative fraction (right panel) of CD8^+^ T_M_ in blood following alemtuzumab (n = 8) or human IgG control mAb (n = 4). Results are shown as mean (+SEM) change from baseline of percentages of baseline absolute counts or percentages of Ki-67. The two-sample WRS test was used to determine the significance of differences in AUC between treatment groups, and the pairwise WRS test to compare cell counts between timepoints (p-values ≤ 0.05 are shown).

To follow up on the observations from the initial pilot study, we performed a larger RM study to determine whether alemtuzumab can affect rebound in RM with lower pre-ART RCVR establishment. To evaluate this, a total of 12 RM (S2 Table) were IV inoculated with 500 IU of the barcoded virus SIVmac239M before starting ART on 7 dpi (Fig 3A) with the goal of limiting the size of the RCVR reservoir to just above a threshold that ensured all untreated RM manifest SIV rebound within 3 weeks of ART release, as previously described [38]. Of note, we used the barcoded SIVmac239M viral stock, which contains up to 10,000 phenotypically identical variants, to facilitate the estimation of reactivation rates of rebounding viral clonotypes, as previously described [39,40]. Following SIV infection, plasma SIV RNA peaked ~10 dpi before declining below 15 SIV RNA copes/ml in all RM by 42 dpi (Fig 3B). Plasma viral load dynamics and the levels of cell-associated viral loads 3-4 days post ART initiation were statistically equivalent between alemtuzumab-treated RM and controls (Fig 3B and 3C).

At 42 weeks pi, after stable ART suppression for ~36 weeks, RMs received 4 weekly doses of alemtuzumab (n = 8) or humanized control IgG (n = 4) at 5mg/kg IV. As observed in the pilot study, alemtuzumab treatment resulted in substantial depletion of CD4^+^ T cells in blood, including >95% depletion of T_N_ and T_M_ subsets (i.e., T_CM_, T_TrM_ and T_EM_) (Figs 3D and 3E and S5A). CD4^+^ T cell depletion was generally transient and accompanied by a rapid increase in CD4^+^ T_M_ proliferation. Of note, CD4^+^ T_N_ reconstitution was substantially slower and less complete than that of CD4^+^ T_M_, as reconstitution of circulating T_N_ in RM is primarily driven by thymic output, which wanes in adulthood [41,42]. To determine the extent of CD4^+^ T cell depletion in tissues, LN sections were obtained before and ~2-weeks after the last dose of alemtuzumab and the frequency of CD4^+^ T cells per unit area of LN section was quantified by immunohistochemistry. Alemtuzumab induced a significant reduction in CD4^+^ T cell densities within the T cell zone of LNs but interestingly, depletion was modest and not significant in B cell follicles and medullary cords (Figs 3F and S6). However, as the LN biopsy was obtained ~2 weeks after the last dose of alemtuzumab, these data may not represent the nadir of CD4^+^ T cell depletion in the LN due to the potential for immune reconstitution occurring prior to when the sample was taken, including through homeostatic proliferation. These data indicate that alemtuzumab can substantially reduce CD4^+^ T cell density in LN of SIV^+^ RM on ART, although depletion was incomplete. Alemtuzumab also had a substantial impact on CD8^+^ T cell dynamics, inducing >95% depletion of CD8^+^ T_M_, which was accompanied by rapid proliferation and reconstitution (Fig 3G). We also observed massive NK cell and B cell depletion, with alemtuzumab having a greater impact on NK cells (>97% depletion) in comparison to B cells (>85% depletion) in the blood (S5C and S5D Fig). Overall, these data confirm the effectiveness of alemtuzumab to deplete CD52-expressing lymphocytes during ART-suppressed SIV infection.

### Effects of alemtuzumab on SIV dynamics during chronic ART

**Fig 4:**
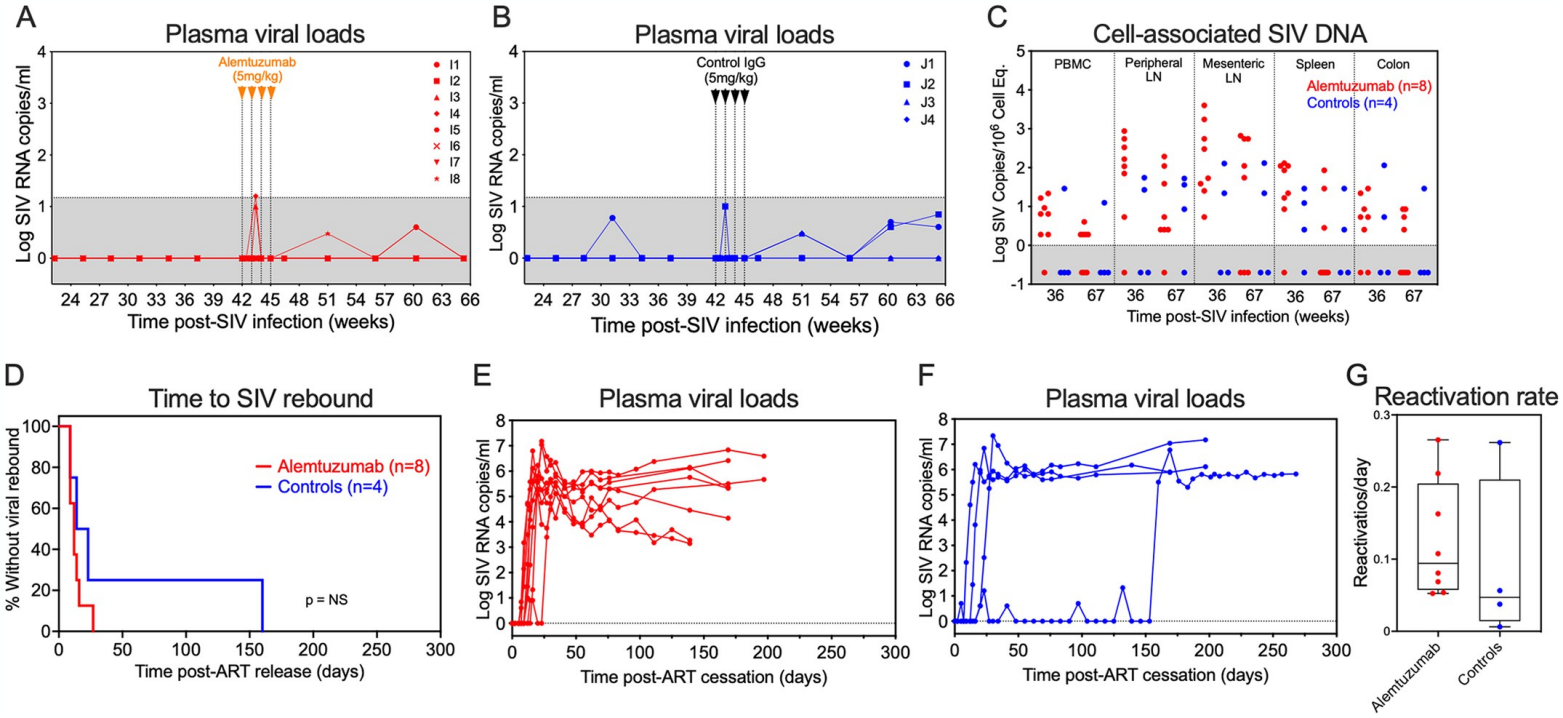
Alemtuzumab treatment during full ART suppression fails to delay post-ART SIV rebound dynamics. (A) Individual plasma viral load (pvl) profiles monitored by high-sensitivity assay (LOD of 1 RNA copy/ml) following alemtuzumab or (B) human IgG control mAb (n = 4) during ART. Values below assay threshold are indicated by the gray area. (C) Comparison of SIV DNA levels in PBMC, peripheral lymph node (LN), mesenteric LN, spleen and colon (copies per 10^6^ cell equivalents) between alemtuzumab-treated RM (n = 8) and human IgG-treated controls (n = 4) at 36- and 67-weeks pi. Assay threshold sensitivity varied as a function of the number of cells available for analysis; values below threshold are indicated by the gray area. (D) Kaplan-Meier analysis of SIV rebound kinetics in RMs treated with alemtuzumab (n = 8) or human IgG control (n = 4). (E and F) Individual pvl profiles of RM in each treatment group following ART release at 533 dpi. The dotted line indicates a pvl threshold of 15 RNA copies/ml. (G) Comparison of SIVmac239M clonal reactivation rates in plasma by high-throughput sequencing after ART cessation between RM in each treatment group.

To characterize the effects of alemtuzumab on SIV dynamics, we initially monitored plasma SIV RNA with a high sensitivity assay (limit of detection = 1 SIV RNA copy/ml) to look for treatment-related pVL blips that might not be apparent with the standard pVL assay threshold of 15 SIV RNA copies/ml and saw no overall difference in the number and magnitude of viral blips between alemtuzumab-treatment and control RM (Fig 4A and 4B). We also assessed whether alemtuzumab had any durable impact on infected cell frequency by quantifying cell-associated SIV DNA in blood, peripheral LN, mesenteric LN, spleen and colon at 36- and 67-weeks post-SIV infection (corresponding to 6 weeks prior to and 31 weeks post the first dose of alemtuzumab, respectively) and found no significant difference between alemtuzumab-treated RM and controls (Figs 4C and S7). Finally, after 75 weeks of ART administration, ART was discontinued to determine the impact of alemtuzumab treatment on the dynamics of viral rebound. All 8 alemtuzumab-treated RM and 3 of 4 control RM manifested viral rebound within 27 days of ART release, with the last control RM showing SIV rebound by day 160 (Fig 4D and 4F). Overall, there was no significant difference in the time to measurable rebound viremia between alemtuzumab-treated RM and controls. Additionally, we used the proportional representation of individual SIVmac239M barcode-defined clones in relation to total rebound viremia at each timepoint to estimate the average clonal viral reactivation rates in individual RM with post-ART viremia [39] and found no significant difference in the average rates of viral clonotypic reactivation between alemtuzumab-treated RM and controls (Fig 4G). Collectively, these data suggest that at the levels of virus infection achieved in these RM prior to ART initiation, alemtuzumab-mediated lymphocyte had no durable effect on the size of the RCVR.

### Effects of alemtuzumab at the time of ART initiation on SIV dynamics

**Fig 5:**
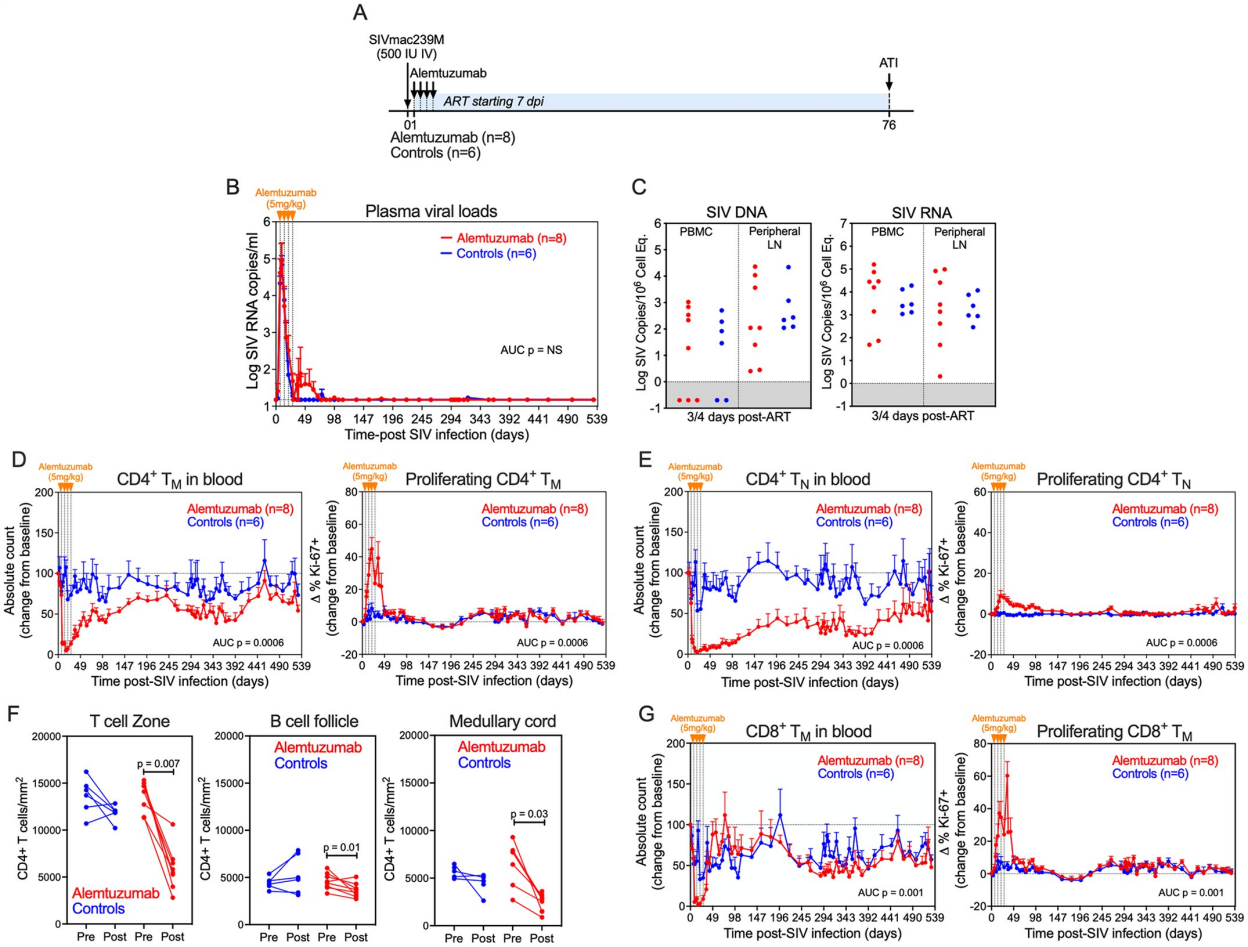
Alemtuzumab at time of ART initiation depletes CD4^+^ and CD8^+^ T cells and impedes plasma viral load suppression. (A) Schematic representation of the study protocol showing SIVmac239M infection, ART initiation 7 dpi and alemtuzumab (n = 8) or human IgG control mAb (n = 6) treatment at 7, 14, 21, 28 dpi. (B and C) Mean (+SEM) plasma viral load profiles and comparison of SIV DNA (left panel) and SIV RNA (right panel) levels in PBMC and peripheral lymph node (LN) (copies per 10^6^ cell equivalents) 3-4 days post ART initiation of alemtuzumab-treated RM (n = 8) versus human IgG-treated controls (n = 6). Assay threshold sensitivity varied as a function of the number of cells available for analysis; values below threshold are indicated by the gray area. (D and E) Change in absolute counts (left panel) and change in the proliferative fraction (right panel) of CD4^+^ memory (T_M_) and naïve (T_N_) T cell subsets in blood following alemtuzumab (n = 8) or human IgG control mAb (n = 6). Results are shown as mean (+SEM) change from baseline of percentages of baseline absolute counts or percentages of Ki-67. (F) Quantification of the number of CD4^+^ cells per 1 × 10^5^ cells in T cell zone, B cell follicle and medullary cords of in LN before and after alemtuzumab treatment. LN biopsies were obtained between 7–13 days after the last dose of alemtuzumab. Each data point represents the average number of CD4^+^ cells derived from quantitative measures from 2–3 LN sections from a single time point from an individual RM. (G) Change in absolute counts (left panel) and change in the proliferative fraction (right panel) of CD8^+^ T_M_ in blood following alemtuzumab (n = 8) or human IgG control mAb (n = 6). Results are shown as mean (+SEM) change from baseline of percentages of baseline absolute counts or percentages of Ki-67. The two-sample WRS test was used to determine the significance of differences in AUC between treatment groups, and the pairwise WRS test to compare cell counts between timepoints (p-values ≤ 0.05 are shown).

Next, we performed an additional study to determine whether alemtuzumab would be more effective at disrupting RCVR dynamics if lymphocyte depletion was performed at the time of ART initiation. We hypothesized that lymphocyte depletion at the time of starting ART would limit SIV reservoir seeding by potentially eliminating both infected cells and uninfected potential target cells and therefore interrupt the establishment of a stable RCVR. To evaluate this, a total of 14 RM (S3 Table) were IV inoculated with 500 IU of the barcoded virus SIVmac239M before starting ART 7 dpi (Fig 5A). RMs received 4 weekly doses of alemtuzumab (n = 8) or humanized control IgG (n = 6) at 5mg/kg IV starting 7 dpi. Following SIV infection, plasma SIV RNA peaked at ~10 dpi before declining below threshold (15 SIV RNA copes/ml) in most RM by 42 dpi (Fig 5B). However, 3 of 8 alemtuzumab-treated RM had a delay in virus suppression compared to control RM (S8 Fig). Quantification of cell-associated viral loads in blood and peripheral LN at 3/4 days-post ART initiation (time of peak viremia) revealed no significant difference between alemtuzumab-treated and control RMs (Fig 5C). Alemtuzumab treatment at the time of ART initiation also resulted in substantial depletion of CD4^+^ T cell subsets (T_M_, T_CM_, T_TrM_, T_EM_ and T_N_) in blood (Figs 5D and 5E and S9A). CD4^+^ T cell depletion was generally transient and accompanied by a rapid increase in CD4^+^ T_M_ proliferation that peaked ~3 weeks after the first alemtuzumab infusion. Quantification of CD4^+^ cells in LN obtained within 7–13 days after the last dose of alemtuzumab revealed treatment at time of ART initiation induced significant depletion of CD4^+^ within the T cell zone, B cell follicles and medullary cords (Fig 5F). Finally, longitudinal analyses of CD8^+^ T cells, NK cells and B cells in blood revealed alemtuzumab treatment at time of ART initiation also had a profound impact on these immune cell subsets. Alemtuzumab induced a rapid depletion of CD8^+^ T_M_ in blood, which was accompanied by CD8^+^ T_M_ proliferation and reconstitution (Fig 5G). NK cell and B cell depletion was also observed in alemtuzumab-treated RM (S9B and S9C Fig).

**Fig 6:**
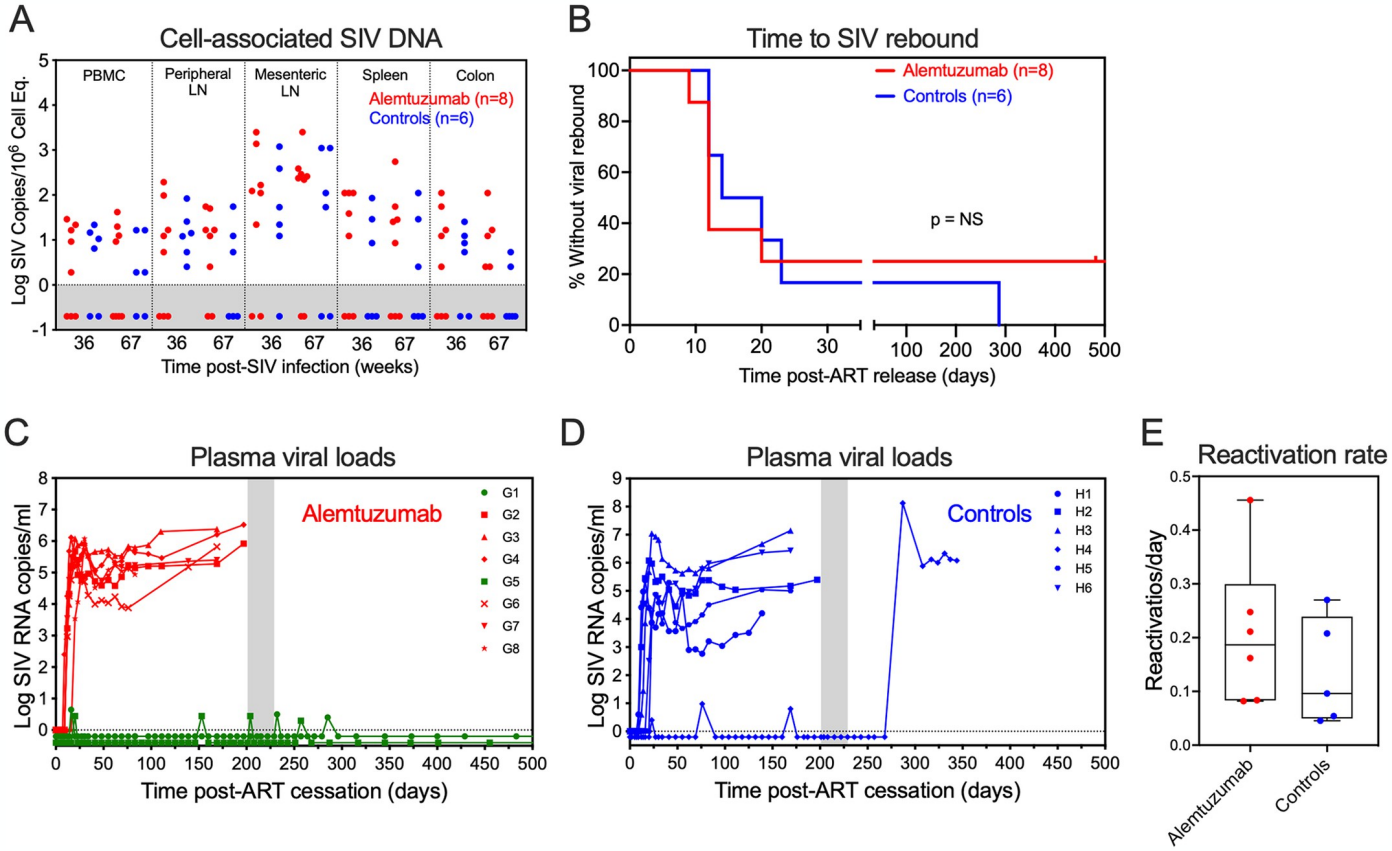
Alemtuzumab at time of ART initiation had a limited impact on SIV rebound dynamics. (A) Comparison of SIV DNA levels in PBMC, peripheral lymph node (LN), mesenteric LN, spleen and colon (copies per 10^6^ cell equivalents) between alemtuzumab-treated RM (n = 8) and human IgG-treated controls (n = 6) at 36- and 67-weeks pi. Threshold sensitivity varied as a function of the number of cells available for analysis; values below threshold are indicated by the gray area. (B) Kaplan-Meier analysis of SIV rebound kinetics in RMs treated with alemtuzumab (n = 8) or human IgG control (n = 6). (C and D) Individual plasma viral load (pvl) profiles of RM in each treatment group following ART release at 533 dpi. The dotted line indicates a pvl threshold of 15 RNA copies/ml. The gray bar indicates CD8 depletion with 50mg/kg of mAb MT807R1 on days 200, 214 and 228 post-ART release. RM G1 and G5 with no post-ART rebound viremia are shown in green. (E) Comparison of SIVmac239M viral barcode clonal reactivation rates in plasma by high-throughput sequencing after ART cessation between RM in each treatment group.

**Fig 7:**
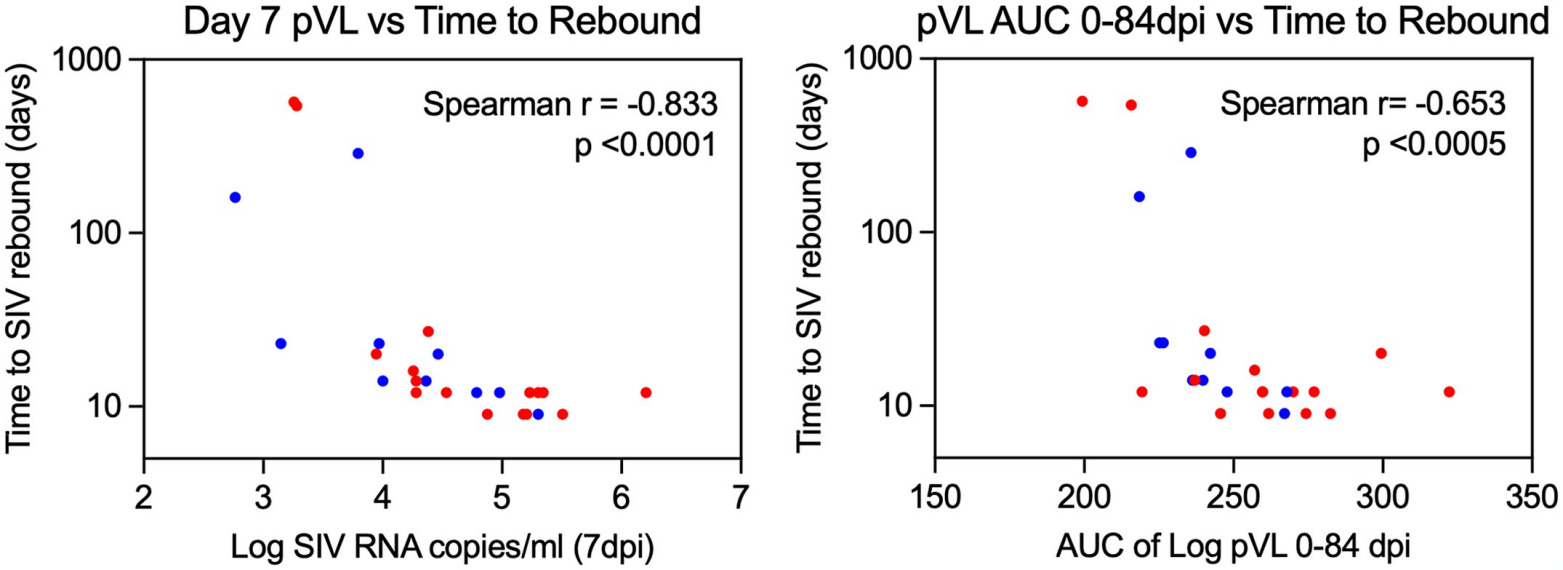
Time to SIV rebound correlates with levels of virus production at time of ART initiation. Scatterplots of time to SIV rebound versus plasma viral loads (pvl) at time of ART on 7 dpi (left panel) and area under curve (AUC) of pvl between 0–84 dpi (right panel) in RM infected with 500IU of SIVmac239M, placed on ART 7 dpi and treated with alemtuzumab at time of ART or during stable ART suppression (n = 16); or a human IgG control antibody at time of ART or during stable ART suppression (n = 10). Spearman rank correlation coefficient r with unadjusted p values testing association between paired samples are shown.

To determine whether alemtuzumab treatment at the time of ART had any durable impact on infected cell frequencies we quantified cell-associated viral loads in blood, peripheral LN, mesenteric LN, spleen and colon at 36- and 67-weeks post-SIV infection and found no significant difference in levels of SIV DNA between alemtuzumab-treated RM and controls (Figs 6A and S10). This suggests that at the levels of lymphocyte depletion achieved, alemtuzumab treatment at time of ART failed to substantially interrupt the dynamics of reservoir seeding or increase the rate of decay of persistently infected cells. After 75 weeks of ART administration, ART was discontinued to determine the impact of alemtuzumab treatment on the rate and extent of SIV rebound. Here, 6 of 8 alemtuzumab-treated RM and 5 of 6 control RM manifested viral rebound within 23 days of ART release, with no substantial difference in time to SIV rebound observed between alemtuzumab-treated RM and controls (Fig 6B–6D). We also found no difference in the average rates of viral clone reactivation between treatment groups (Fig 6C). Instead, when we combine RM that were challenged with the same infectious dose of SIVmac239M and started on ART 7 dpi (Study 2 and 3), we found time to rebound was associated with pVL at time of ART initiation (Fig 7), suggesting alemtuzumab treatment, either at the time of ART or during full ART suppression, had no long-term impact on the stability of the RCVR in most RM with typical levels of early viral replication.

Two alemtuzumab-treated RM (G1 and G5) and a control RM (RM H4) did not manifest SIV rebound initially post-ATI. Of note, RM G1 and G5 had the lowest levels of pre-ART peak pVL among alemtuzumab-treated RM at 1,900 and 2,600 SIV RNA copies/ml, respectively, while RM H4 peaked at 21,000 SIV RNA copies/ml. To determine whether the lack of rebound was associated with CD8^+^ cell-mediated control, we administered MT807R1 starting 200 days after ART release. Following CD8^+^ cell depletion, virus rebound was observed in the control RM (H4) at 287 days post-ART but the two alemtuzumab-treated RM continued to remain aviremic up to 500 days post-ATI (Fig 6C and 6D). These data suggest that the 2 RM with no post-ART viremia were not immune controllers of a small RCVR but rather had experienced full decay of the RCVR prior to ART release and thus were presumptively cured of SIV infection.

**Fig 8:**
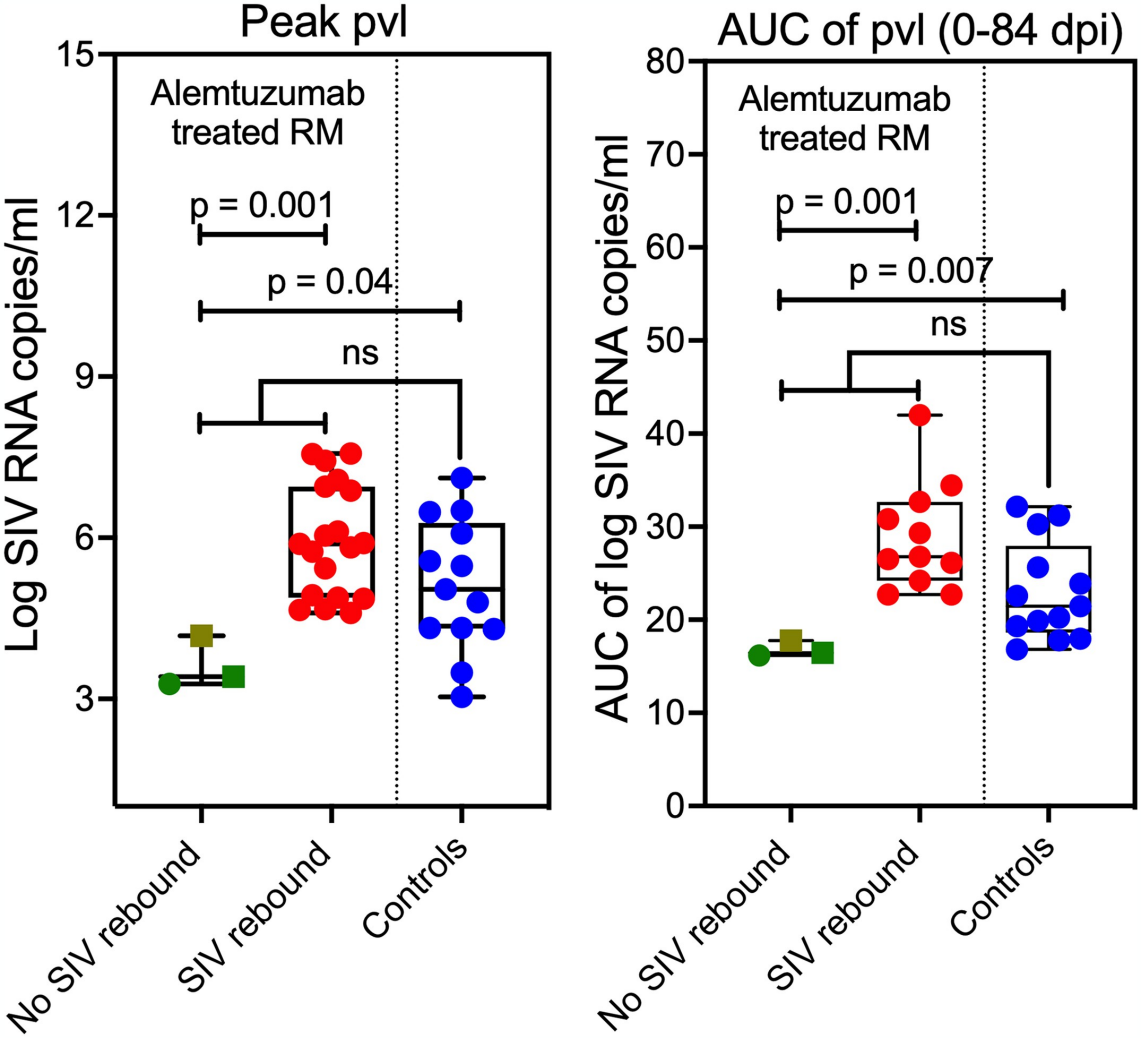
Alemtuzumab-treated RM without post-ART rebound viremia had relatively low virus production at time of ART initiation. Comparison of peak plasma viral load (pvl) and total viral burden, as measured by AUC of pvl between 0–84 dpi, in alemtuzumab-treated RM and no post-ART rebound viremia (n = 3) relative to alemtuzumab-treated RM with post-ART rebound viremia (n = 19). Note that all control RM (n = 14) manifested SIV rebound following ART cessation. The two-sample WRS test was used to determine the significance of differences between treatment and outcome groups (p-values ≤ 0.05 are shown).

Next, we explored the characteristics of the alemtuzumab-treated RM with no post-ART rebound viremia, including in RM G1 and G5 that received alemtuzumab at time of ART initiation and RM D6 from the pilot study that was treated during full ART suppression, and found pVL at peak and pVL area under curve (AUC) between 0–84 dpi were significantly lower in these 3 RM relative to the 19 alemtuzumab-treated RM that manifested SIV rebound (Fig 8). Interestingly, IgG control-treated RM with similarly low levels of pre-ART SIV replication all manifested rebound viremia. Of note, the lack of rebound was not associated with differential levels of CD4^+^ T_M_ depletion as this was equivalent across alemtuzumab-treated RM (S11 Fig). Additionally, the absence of rebound was not associated with the presence of protective MHC-I alleles associated with control of SIV replication (S1 and S3 Tables). Collectively, these data suggests that when virus replication prior to ART is substantially constrained, alemtuzumab depletion may further disrupt the low levels of RCVR that were established. However, once RCVR are established beyond that threshold, alemtuzumab had no measurable efficacy at preventing SIV rebound, likely due to insufficient depletion of infected cells and potential CD4 targets in tissues as well as the massive T cell proliferation driven by immune reconstitution.

## Discussion

CD4^+^ T cells containing replication-competent HIV proviruses that persist during ART and give rise to recrudescent viremia after ART cessation (operationally defined in this study as RCVR) represent the primary barrier to curing HIV infection. In this study, we assessed whether pan-lymphocyte depletion with alemtuzumab can reduce or eliminate the RCVR in SIV-infected RM when is administered at the time of ART initiation, 7 dpi, or during chronic ART administration. In contrast to strategies which seek to specifically target only infected cells which spontaneously or following “latency reversing agent” treatment express viral antigens, alemtuzumab treatment was used in this study to inclusively deplete both infected cells and potential target non-infected CD4^+^ T cells, along with other populations. In this proof-of-concept study, we sought to test the potential of extensive cell depletion administered during different phases of infection to impact the RCVR and viral rebound, rather than to establish a specific treatment with near term clinical translatability. Among 22 RM treated with alemtuzumab during chronic ART or at the time of ART initiation, we observed >95% depletion of CD4^+^ T cells in the peripheral blood. Additionally, CD4^+^ T cell depletion in LNs was substantial but not complete. However, despite the massive loss of CD4^+^ T cells, we observed no quantifiable effects of alemtuzumab on the RCVR as assessed by cell-associated viral loads during ART and SIV rebound dynamics following ATI in almost all (19 of 22) alemtuzumab-treated RM. The inability of alemtuzumab to permanently disrupt the RCVR may be due to a RCVR that even with early ART, was too large and/or lymphocyte depletion that was not complete enough to facilitate a cure or even delay viral rebound.

We generally observed higher levels of CD4^+^ T cell depletion within the T cell zone of LN with relative sparing of CD4^+^ T cells in B cell follicles, particularly in RM that received alemtuzumab during chronic ART. This is notable as HIV/SIV-infected follicular helper CD4^+^ T cells (T_FH_) resident within B cell follicles substantially contribute to residual levels of viral nucleic acid during ART [43]. This has been associated with the relative exclusion of most anti-viral effector CD8^+^ T cell responses, which often lack expression of the chemokine receptor CXCR5 required for follicular entry [44–46]. Thus, CD8^+^ T cell responses are often limited in their ability to target and control productive HIV/SIV infection of CD4^+^ T_FH_ within B cell follicles [47]. Hence, incomplete depletion within B cell follicles could allow residual latently-infected CD4^+^ T_FH_, which are protected from effective CD8^+^ T cell immunity, to serve as sites of early viral reactivation and spread once ART is withdrawn. Of note, HIV RNA^+^ T_FH_ have been shown to persist for > 4 years in LN of PWH treated in Fiebig I/II, suggesting T_FH_ reservoirs are established very early during primary infection [48].

While we did not characterize the extent of CD4^+^ T cell depletion in mucosal compartments, there is evidence to suggest that alemtuzumab is able to deplete lymphocytes in gut-associated lymphoid tissues [49]. However, clinical data in humans suggests that some tissue resident populations may still be spared from depletion based on the observation that alemtuzumab treatment is not associated with increased risk for severe opportunistic infections (OIs) [50,51]. Even PWH that received alemtuzumab as part of pre-renal transplant conditioning showed no substantial increase in OIs despite delayed CD4^+^ T cell reconstitution [52]. This relative protection from OIs has been attributed to the maintenance of mucosal immunity due to the preservation of tissue resident T_EM_ that survive alemtuzumab depletion [50]. Hence, residual CD4^+^ T cells with RCVR in mucosal compartments could have limited the effectiveness of alemtuzumab therapy. However, this also suggests that targeting these alemtuzumab-resistant populations may be essential for ensuring complete RCVR elimination following lymphocyte depletion. This is critical as incomplete CD4^+^ T cell depletion, together with a high drive for homeostatic proliferation as a result of CD4^+^ T cell reduction, all likely contributed to the ability of the RCVR to reconstitute after alemtuzumab treatment. This was highlighted by the dramatic increases in proliferating CD4^+^ T_M_ we observed in blood following alemtuzumab treatment. As proliferation of latently-infected cells can occur without proviral gene expression [53], it suggests that homeostatic proliferation can support RCVR reconstitution without virus expression resulting in clearance of infected cells by immune recognition and/or viral cytopathic effects. Indeed, if expanded clones of CD4^+^ T cells harboring clonally integrated SIV were established prior to alemtuzumab treatment, so long as some of these cells survived the depletion, the post-depletion homeostatic proliferation response could reconstitute or even expand the infected clone population. This is consistent with observations that CD4 depleting mAbs in SIV-infected RM on ART failed to durably reduce levels of SIV DNA in blood and lymphoid tissues [54,55]. These findings are also consistent with the only transient decline in HIV DNA levels in blood observed in PWH that received lymphocyte-depleting immunosuppression prior to kidney transplantation [56]. The negligible effect of these lymphocyte depleting therapies on viral reservoir dynamics clearly demonstrates that the incomplete depletion levels of the cells harboring the RCVR typically achieved by such approaches can reconstitute to levels established prior to depletion, when there is a strong drive for cellular proliferation.

A surprising observation from our study was the altered kinetics of virologic suppression observed when alemtuzumab was administered at the time of ART initiation. Indeed, we had hypothesized that lymphocyte depletion at the time of ART initiation would limit the size and distribution of the RCVR due to the depletion of CD4^+^ viral target cells. However, instead of an accelerated decline in pVL, the kinetics of SIVmac239 suppression was variable with 3 of 8 alemtuzumab-treated RM manifesting delayed viral suppression compared to control RM, likely associated with the activation and proliferation of CD4^+^ T cells and/or a disruption in anti-viral immune responses due to the depletion of CD8^+^ T cells and NK cells. Indeed, experimental CD8^+^ depletion in SIV-infected RM before or at time of ART initiation was also shown to delay pVL suppression [57]. However, despite the extended period of viremia in these RM, there was no long-term impact on levels of SIV DNA across multiple tissue compartments in RM that received alemtuzumab at the time of ART relative to controls. This would indicate that circulating lymphocytes at the time of ART, majority of which were susceptible to alemtuzumab-mediated depletion, do not play a primary role in RCVR persistence. In contrast, our data suggests that cells with RCVR in tissues, that were relatively resistant to alemtuzumab, were sufficient to ensure RCVR stability. This may explain why an individual who received ART within 10 days of HIV exposure manifested HIV rebound post-ART despite an extended period of undetectable viral nucleic acid [58]. Also, RM that received ART as early as 3 dpi following mucosal challenge and maintained on ART for 24 weeks, manifested SIV rebound despite no detectable viremia prior to ART [59], which suggests that RCVR established early in mucosal tissues, in the absence of systemic spread, can persist for an extended period of time. Of note, by varying the timing of ART initiation in RM infected with the barcoded virus SIVmac239M, we have recently observed the seeding of the RCVR appears to be saturable within 9 days of intravenous inoculation despite subsequent ongoing accumulation of SIV DNA^+^ cells in blood and tissues [40], likely due to shortened survival of cells infected in an increasingly proinflammatory milieu through the course of primary infection and development of antiviral immune responses. The RCVR is not only established early after infection but also rapidly stabilizes such that further increases in SIV DNA levels are no longer directly predictive of rebound.

While these studies suggest that persistence of even a limited fraction of the RCVR can support eventual viral rebound, we did observe three alemtuzumab-treated RM that failed to manifest rebound viremia during extended follow up after ART withdrawal. As these animals had the lowest levels of pre-ART SIV replication, it suggests that alemtuzumab may have been effective against the early and presumably smaller RCVR established in these RM. However, whether the lack of rebound was directly associated with alemtuzumab-mediated depletion of all of the RCVR or an inherent instability in RCVR previously observed following early ART initiation in this RM model remains unclear [38, 40]. Addressing this in future nonhuman primate (NHP) studies will help improve our understanding of RCVR biology and allow for determining the requirements for more clinically feasible RCVR targeting interventions to achieve a meaningful benefit.

The present results make clear that such studies would require an even greater level of infected and target cell depletion in tissues, than observed here with alemtuzumab. To achieve this, antibody engineering techniques could be applied to enhance the effector functions of lymphocyte-depleting mAbs to improve the targeting of tissues-resident populations [60,61]. Cytokines such as IL-15 could also be used to improve the depletion efficiency of mAbs via the activation of NK cells to increase antibody-dependent cellular cytotoxicity [62]. An alternative strategy could be to combine lymphocyte-depleting mAbs with antiproliferative agents to limit the homeostatic expansion and reconstitution of CD4^+^ T cells that harbor RCVR virus. Of note, CD4^+^ T cell reconstitution was shown to be substantially delayed in cynomolgus macaques that received the antiproliferative agent mycophenolate mofetil (MMF) after alemtuzumab treatment relative to animals that did not receive MMF [37]. We also reported that mTOR inhibition with rapamycin can substantially reduce levels of CD4^+^ T cell proliferation in SIV-infected RM on ART [63]. Whether MMF, rapamycin or other antiproliferative agents can be used to enhance the efficacy of lymphocyte depleting mAbs to permanently disrupt RCVR stability in RM remains to be determined.

In conclusion, we used a well-characterized NHP model of ART-suppressed SIVmac239 infection of RM to assess the impact of pan-lymphocyte depletion with alemtuzumab on RCVR dynamics. Our results demonstrate that the RCVR can reconstitute following substantial depletion of targeted populations such that laboratory measures of viral persistence and viral rebound dynamics remain relatively unchanged. Our studies highlight the current limitation of lymphocyte depletion strategies in facilitating the permanent disruption of RCVR, particularly once the RCVR is established above thresholds that ensure consistent virologic rebound.

## Materials and methods

### Ethics statement

All studies were conducted with the approval of Oregon National Primate Research Center’s Animal Care and Use Committee (TR01_IP00001053), under the standards of the US National Institutes of Health Guide for the Care and Use of Laboratory Animals.

### Rhesus macaques

A total of 35 purpose-bred male and female RMs (*Macaca mulatta*) of Indian genetic background were used for these experiments. All RM were specific pathogen-free as defined by being free of cercopithecine herpesvirus 1, D-type simian retrovirus, simian T-lymphotropic virus type 1, and *Mycobacterium tuberculosis*. MHC-1 genotyping for common Mamu alleles such as Mamu-A*01/-A*02 and Mamu-B*08/-B*17 was performed by sequence-specific priming PCR, essentially as previously described [64]. Prior to SIV infection, all RM were screened for CD52 expression on erythrocytes using an agglutination assay as described previously [37]. In the pilot study, 9 male RM (3–8 years of age) with no alemtuzumab-mediated erythrocyte agglutination were IV inoculated with 2 IU of SIVmac239X before starting ART consisting of daily subcutaneous injections of 5.1 mg kg^−1^ d^−1^ tenofovir disoproxil, 40 mg kg^−1^ d^−1^ emtricitabine and 2.5 mg kg^−1^ d^−1^ dolutegravir in a solution containing 15% (v/v) kleptose at pH 4.2, as previously described [65], on 12 dpi through 530 dpi. In addition, 6 RM received alemtuzumab (Genzyme, Cambridge MA) at 5 mg/kg IV on 294, 301, 308 and 322 dpi. The RM without measurable viremia was treated with anti-CD8α mAb, MT807R1 (Nonhuman Primate Reagent Resource) at 252 days post-ART, administered SubQ at 10 mg/kg on day 0 then IV at 5 mg/kg on days 3, 7, and 10.

An additional 26 RM (17 males, 9 females; 3–5 years of age) were also screened for CD52 expression using an agglutination assay and flow cytometry. For safety, RM with erythrocyte agglutination and confirmed CD52 staining on red blood cells were placed in the control groups. However, experimental arms (i.e., early intervention or late intervention) were also balanced based on RM age and sex (S2 and S3 Tables). RM were IV inoculated with 500 IU of the barcoded SIVmac239M before starting ART on 7 dpi through 532 dpi. One group of RM received 4 doses of alemtuzumab (n = 8) or control human IgG1 (52H5) mAb, (Nonhuman Primate Reagent Resource) (n = 6) at 5mg/kg on 7, 14, 21 and 28 dpi. Another group of RM received 4 doses of alemtuzumab (n = 8) or control human IgG1 (52H5) mAb (n = 4) on 294, 301, 308, 315 dpi. Of note, RM in the second group were pre-treated with steroids prior to antibody infusion to mitigate adverse reactions. The 2 alemtuzumab-treated and 1 control RM without measurable viremia were treated with MT807R1 (Nonhuman Primate Reagent Resource) administered SubQ at 50 mg/kg at day 0 and IV at 50 mg/kg on days 14 and 28 starting between 201- and 229-days post-ART cessation.

### Viruses

The SIVmac239M challenge stock used in this experiment was produced by transfection of HEK-239T cells and the stock infectivity titer was determined using TZM-bl cells as previously described [39]. The infection derived SIVmac239X challenge stock was generated by expansion in RM PBMC and tittered using TZM-bl cells as previously described [66].

### SIV viral detection assays

Plasma SIV RNA levels were determined using a gag-targeted quantitative real time/digital RT-PCR format assay, essentially as previously described, with 6 replicate reactions analyzed per extracted sample for assay threshold of 15 SIV RNA copies/ml [67]. Ultrasensitive determinations of plasma SIV RNA were measured by concentrating virus from larger volumes of plasma by centrifugation. For ultrasensitive measurements, typically, 1.7 ml of plasma were centrifuged in a refrigerated microfuge (21,000 x g, 1 hr, 4° C) and nucleic acid was extracted from pellets as described [68] and quantitative RT PCR was performed with 12 reactions per extracted sample. Samples that did not yield any positive results across the replicate reactions were reported as a value of “less than” the value that would apply for one positive reaction out of 12 [67]. As performed, the ultrasensitive assay provided a threshold sensitivity of 1 copy/ml plasma for a 1.7 ml sample. Quantitative assessment of SIV DNA and RNA in cells and tissues was performed using gag targeted nested quantitative hybrid real-time/digital RT-PCR and PCR assays, as previously described [67,69]. SIV RNA or DNA copy numbers were normalized based on quantitation of a single copy rhesus genomic DNA sequence from the CCR5 locus from the same specimen to allow normalization of SIV RNA or DNA copy numbers per 10^6^ diploid genome cell equivalents, as described [70]. Ten replicate reactions were performed with aliquots of extracted DNA or RNA from each sample, with two additional spiked internal control reactions performed with each sample to assess potential reaction inhibition. Samples that did not yield any positive results across the replicate reactions were reported as a value of “less than” the value that would apply for one positive reaction out of 10. Threshold sensitivities for individual specimens varied as a function of the number of cells or amount of tissue available and analyzed.

### Barcode sequencing

Barcode sequencing was performed as previously described [39,71]. Briefly, RNA was isolated from plasma using QIAamp Viral RNA mini kit per manufacturer’s instructions. cDNA was then synthesized from the extracted DNA using Superscript III reverse transcriptase (Invitrogen) and a reverse primer (Vpr.cDNA3: 5’-CAG GTT GGC CGA TTC TGG AGT GGA TGC-3’). qRT-PCR was used to quantify the cDNA using the primers VpxF1 5’-CTA GGG GAA GGA CAT GGG GCA GG-3’ at 6082–6101 and VprR1 5’-CCA GAA CCT CCA CTA CCC ATT CATC-3’ at 6220–6199 and a fluorescently labeled probe (ACC TCC AGA AAA TGA AGG ACC ACA AAG GG). Known quantities of the viral template were then PCR amplified with the same VpxF1 and VprR1 primers but with MiSeq adaptors directly synthesized onto the primers. Reactions were prepared using High Fidelity Platinum Taq per the manufacturer’s instructions, using primer VpxF1 and VprR1 with the following conditions: 94°C for 2m followed by 40 cycles of 94°C, 15s; 60°C, 90s; 68°C, 30s with final extension of 68°C for 5m. Following PCR-cleanup, amplicons were pooled and sequenced directly on MiSeq instrument (Illumina).

### Immunohistochemistry

IHC was performed using a biotin-free polymer approach (Golden Bridge International, Inc.) on 5-μm tissue sections mounted on glass slides, which were dewaxed and rehydrated with graded alcohols to double-distilled H_2_O. Heat-induced epitope retrieval was performed by heating sections in 0.01% citraconic anhydride containing 0.05% Tween-20 in a pressure cooker set at 110°C for 15 min. For IHC of myeloid cells, slides were loaded on an IntelliPATH autostainer (Biocare Medical) and stained with optimal conditions determined empirically consisting of a blocking step using blocking buffer for 30 min, an endogenous peroxidase block using 1.5% (v/v) H_2_O_2_ in TBS (pH 7.4) for 5 min, incubation with mouse anti-CD68 (1:200; clone KP1, Biocare CM003C), mouse anti-CD163 (1:600; clone 10D6; Thermofisher (MA5-11458)) and rabbit monoclonal anti-CD4 (1:200; clone EPR6855; Abcam ab133616) diluted in blocking buffer for 1 h at room temperature, washed with TBS containing 0.05% Tween-20 (TBS–Tw) and detection using a biotin-free polymer approach consisting of Rabbit Polink-1 HRP (Golden Bridge International, Inc.) for 20 min at room temperature followed by Mouse Polink-2 AP (Golden Bridge International, Inc.) for 20 min at room temperature. Sections were washed and first incubated with Impact DAB (3,3′-diaminobenzidine; Vector Laboratories) to develop the CD4, washed and developed with Warp Red (Biocare Medical, Inc.) to develop macrophage/myeloid cells and to mask the faint CD4 expressed on APCs to distinctly identify CD4^+^ T cells from myeloid cells.

All slides were washed in ddH_2_O, counterstained with haematoxylin, mounted in Permount (Fisher Scientific) and scanned at high magnification (× 20) using a whole-slide scanning microscope (Aperio AT2 System, Aperio Technologies), yielding high-resolution data from the entire tissue section. Anatomic regions within the LNs were assigned to B cell follicles (BCFs), medullary cords (MCs), and the paracortical T cell zone (TCZ) based off the staining pattern of CD4 and CD68^-^CD163^-^. The number of CD4 T cells and CD68/163 positive macrophages were analyzed by the Multiplex IHC v3.0.4 module of Halo (v3.5.3577 Indica Software), and normalized to positive cells per mm^2.

### Immunophenotyping

To determine the phenotype of lymphocyte populations in whole blood were stained as previously described [42,72,73]. Polychromatic (8–14 parameter) flow-cytometric analysis was performed on an LSR II BD instrument using Pacific blue, BUV395, BUV495, BUV737, BUV805, BV421, BV510, BV570, BV605, BV650, BV711, BV786, FITC, PE, PE-Texas red (PE-CF594), PE-Cy7, PerCP-Cy5.5, APC, APC-Cy7, and Alexa 700 as the available fluorescent parameters. Instrument setup and data acquisition procedures were performed as previously described [42,72,73]. List mode multiparameter data files were analyzed using the FlowJo software program (BD Biosciences). Lymphocytes were distinguished as follows: T cells are CD3^+^, CD20^-^ small lymphocytes, B cells are small lymphocytes that are CD3^-^ and either CD20^+^ or CD79a^+^, NK cells are CD3^-^, CD20^-^, HLA-DR-, CD8α^+^ small lymphocytes and myeloid cells are CD3^-^, CD20^-^, CD8α^-^, HLA-DR^+^ mononuclear cells. T cells were further subdivided into memory and naive subsets and Ki-67 high expression was defined using criteria previously described [42,72,73]. Briefly, naïve T cells constitute a uniform cluster of cells with a CD28^moderate^ CD95^low^ phenotype that are CCR7^+^ and CCR5^−^. Memory T cells are a phenotypically diverse memory population that is CD95^high^ or displays one or more of the following non-naive phenotypic features: CD28^−^, CCR7^−^, CCR5^+^. The T_CM_, transitional memory T cell (T_TrM_), and effector memory T cell (T_EM_) components of the memory subset in the blood were further delineated based on the following phenotypic criteria: T_CM_ (CD28^+^, CCR7^+^, CCR5^−^), T_TrM_ (CD28^+^, CCR7^+/−^, CCR5^+^), and T_EM_ (CD28^−^, CCR7^−^, CCR5^dim^). To quantify each subset in tissues, the frequency of the subset within the overall small lymphocyte population was determined. For quantification of immune subsets in peripheral blood, small lymphocyte counts were obtained using an ABX Pentra 60 Hematology Analyzer (Horiba ABX SAS) and, from these values, absolute counts for the relevant subset were calculated based on the subset frequencies within the light scatter–defined small lymphocyte population on the flow cytometer. Baseline values were determined as the average of values at 3 timepoints prior to treatment (SIV infection or first antibody dose). Absolute counts are indicated as percentage of baseline with baseline shown as 100%. Changes in proliferative fraction are indicated as the change in the %Ki-67^+^ (Δ%Ki-67^+^) of the parental subset measured at the designated time points from baseline (0% = no change).

### Antibodies

Combinations of fluorochrome-conjugated monoclonal antibodies used for staining included anti-CD3 (SP34-2: BUV395; BD Biosciences, Custom Bulk 624310 and APC-Cy7; BD Biosciences, Custom Bulk 624072), anti-CD4 (L200: BV786; BD Biosciences, Custom Bulk 624159 and BV510; BD Biosciences, Custom Bulk 624340), anti-CD8α (DK25: Pacific Blue; SK1: BUV737; BD Biosciences, Custom Bulk 624235, BV711; BD Biosciences, Custom Bulk 624148 and BV510; BD Biosciences, Custom Bulk 624144), anti-CD95 (DX2: PE; BioLegend, Custom Bulk 94203 and APC; ebioscience, Custom Bulk 7017-0959-M050 and BUV737; BD Biosciences, Custom Bulk 624231), anti-CD28 (CD28.2: PE-DAZZ; BioLegend, Custom Bulk 93364), anti-CCR5 (3A9: APC; BD Biosciences, Custom Bulk 624076), anti-Ki67 (B56: FITC; BD Biosciences, Custom Bulk 624046 and PE; BD Biosciences, Custom Bulk 624048), anti-CD14 (M5E2: FITC; BioLegend, Custom Bulk 94202), anti-CD16 (3G8: BV650; BD Biosciences, Custom Bulk 93384), anti-HLA-DR (L243: PE-DAZZ; BioLegend, Custom Bulk 93957 and BV510; BioLegend, Custom Bulk 93784), anti-CD20 (2H7: APC-Cy7; BioLegend, Custom Bulk 93924), anti-CCR7 (150503: Biotin; R&D Systems, MAB197 and BV711, BD Biosciences, Custom Bulk 624386), anti-NKG2A (REA110: APC; Miltenyi, 130-095-212 and PE, Miltenyi, 130-095-212), anti-CD56 (MEM-188: PerCP-Cy5.5, Life Technologies, MHCD5618CS3), anti-CD69 (CH/4: PerCP-Cy5.5; Life Technologies, MHCD6918), anti-CD27 (M-T271: BV421; BioLegend, Custom Bulk 86409), anti-CD21 (B-ly4: BV711; BD Biosciences, Custom Bulk 624148), anti-IgD (PE; Southern Biotech, 2030–09), anti-PD-1 (eBioJ105: PerCP-Cy5.5; Life Technologies, Custom Bulk CUST00656), anti-CXCR3 (G025H7: BV510; BioLegend, Custom Bulk 94271), anti-CXCR5 (MU5UBEE: PE; Life Technologies, Custom Bulk CUST03534) anti-CD169 (7–239: PE; BioLegend, Custom Bulk 346004) anti-CD123 (7G3: PerCP-Cy5.5; BD Biosciences, Custom Bulk 624060), anti-CD11c (3.9: APC; Life Technologies, Custom Bulk CUST01764), anti-CD86 (2331 [FUN-1]: BV711; BD Biosciences, Custom Bulk 624316), anti-CD40 (5C3: BV510; BD Biosciences, Custom Bulk 624146), anti-CD80 (L307.4: BUV; BD Biosciences, Custom Bulk 624309), anti-CD127 (HIL-7R-M21: BV605; BD Biosciences, Custom Bulk 624143) and anti-streptavidin (BV421; BD Biosciences, Custom Bulk 624337 and BV605, BD Biosciences Custom Bulk 624342).

### Statistics

Statistical analyses were performed in R 4.3.2 with the package “survival” v3.5–7. Point values are transformed to the log_10_ scale where indicated. For analyses involving multiple timepoints, we calculated the area under the curve (AUC) for each RM and analyzed the resulting values using the Wilcoxon rank-sum (WRS) test. We used two-sample WRS tests for all analyses comparing values across treatment groups, and paired WRS tests for analysis comparing values across pre- and post-treatment timepoints. Time-to-event data were described with Kaplan-Meier estimates and compared between groups using the Kruskal-Wallis test. All tests were conducted with two-sided null hypotheses at significance level P ≤ 0.05.

### Sample size and treatment assignment

Sample size was determined by logistical and resource considerations. Treatment assignments (alemtuzumab vs. control human IgG1 treatment) were conducted prior to infection based on erythrocyte CD52 expression and then further balanced between alemtuzumab at time of ART and alemtuzumab during chronic ART treatment groups based on sex, age and presence of protective MHC alleles. No blinding was possible due to the constraints of working with RM.

## Supporting information

S1 Table Characteristics of RM in the pilot study. The table shows the sex, age at the time of SIV infection, challenge virus, peak plasma viral loads, duration of of ART relative to SIV infection and known protective MHC-1 alleles.
(TIF)

S2 Table Characteristics of RM in the follow up study. The table shows the sex, age at the time of SIV infection, challenge virus, peak plasma viral loads, duration of of ART relative to SIV infection and known protective MHC-1 alleles.
(TIF)

S3 Table Characteristics of RM in the follow up study. The table shows the sex, age at the time of SIV infection, challenge virus, peak plasma viral loads, duration of ART relative to SIV infection and known protective MHC-1 alleles.
(TIF)

S1 Fig Alemtuzumab depletes CD4^+^ T cell subsets in blood and induces homeostatic proliferation. Change in absolute counts (left panels) and change in the proliferative fraction (right panels) of CD4^+^ central memory (T_CM_), transitional memory (T_TrM_) and effector memory (T_EM_) in blood following alemtuzumab (n = 6) or no treatment (n = 3). Results are shown as mean (+SEM) change from baseline of percentages of baseline absolute counts or percentages of Ki-67. The WRS test was used to determine the significance of differences between treatment groups (p-values ≤ 0.05 are shown).
(TIF)

S2 Fig Alemtuzumab depletes CD8^+^ T cells, NK cells and B cells in blood. (A and B) Change in absolute counts and (C and D) change in the proliferative fraction of CD8^+^ memory (T_M_) and naïve (T_N_) T cells in blood following alemtuzumab (n = 6) or no treatment (n = 3). (E) Change in absolute counts of CD3^-^ CD8^+^ NKG2A^+^ NK cells and (F) change in absolute counts of CD20^+^ B cells in blood of alemtuzumab-treated RM (n = 6) versus untreated controls (n = 3). Results are shown as mean (+SEM) change from baseline of percentages of baseline absolute counts or percentages of Ki-67. The WRS test was used to determine the significance of differences between treatment groups (p-values ≤ 0.05 are shown).
(TIF)

S3 Fig Effect of Alemtuzumab on plasma viral loads. Individual plasma viral load (pvl) profiles monitored by a high-sensitivity assay (limit of detection [LOD] of 1 RNA copy/ml) prior to and during alemtuzumab treatment (n = 6) or no treatment (n = 3). The area in gray denotes pvl values below threshold of the standard assay (15 RNA copies/ml).
(TIF)

S4 Fig CD8^+^ T cell depletion did not increase plasma viral loads in post-ART non-rebounder. Individual plasma viral load profiles of the post-ART non-rebounder RM D6 and an SIV elite controller RM monitored by a high-sensitivity assay (limit of detection [LOD] of 1 RNA copy/ml) following treatment with the anti-CD8α depleting antibody MT807R1 at 10mg/kg SubQ on day 0 and 5mg/kg IV on days 3, 7 and 10.
(TIF)

S5 Fig Alemtuzumab depletes CD4^+^ T cells, NK cells and B cells in blood. (A) Change in absolute counts (left panels) and proliferative fraction (right panels) of CD4^+^ memory (T_M_) T cell subsets, including central memory (T_CM_), transitional memory (T_TrM_) and effector memory (T_EM_) in blood of alemtuzumab-treated RM (n = 8) versus human IgG-treated controls (n = 4). (B) Change in absolute counts of CD3^-^ CD8^+^ NKG2A^+^ NK cells and (C) change in absolute counts of CD79a^+^ B cells in blood of alemtuzumab-treated RM (n = 8) versus IgG-treated controls (n = 4). Results are shown as mean (+SEM) change from baseline of percentages of baseline absolute counts or percentages of Ki-67. The WRS test was used to determine the significance of differences between treatment groups (p-values ≤ 0.05 are shown).
(TIF)

S6 Fig Characterizing Alemtuzumab-mediated CD4^+^ T cell depletion in lymph nodes. Representative images of immunohistochemical analysis performed on LN sections obtained from an RM before alemtuzumab (-26 dpi) [top] and 10 days after the last dose of alemtuzumab (41 dpi) [bottom]. Magnified images demonstrate the effect of alemtuzumab on the B cell follicles (BCF), T cell zone (TCZ), and medullary cord (MC) regions of the lymph node. CD4^+^ cells are in brown, while CD68^+^CD163^+^ cells are in maroon. Scale bars: 1mm-100 μm.
(TIF)

S7 Fig Effect of alemtuzumab during full ART suppression on cell-associated viral loads. (A) Comparison of SIV DNA (left panels) and SIV RNA (right panels) in PBMC and (B) peripheral LN (copies per 10^6^ cell equivalents) of alemtuzumab-treated RM (n = 8) and human IgG-treated controls (n = 4). Threshold sensitivity varied as a function of the number of cells available for analysis; values below threshold are indicated by the gray area.
(TIF)

S8 Fig Effect of Alemtuzumab at time ART initiation on virologic suppression. (A) Individual plasma viral load (pvl) profiles of alemtuzumab-treated RM and (B) human IgG control antibody-treated RM up to 16 weeks post-SIVmac239M infection. The dotted line indicates a pvl threshold of 15 RNA copies/ml.
(TIF)

S9 Fig Effect of alemtuzumab at time of ART on CD4^+^ T cells, NK cells and B cells in blood. (A) Change in absolute counts (left panel) and proliferative fraction (right panel) of CD4^+^ memory (T_M_) subsets, including central memory (T_CM_), transitional memory (T_TrM_) and effector memory (T_EM_) in blood of alemtuzumab-treated RM (n = 8) versus human IgG-treated controls (n = 6). (B) Change in absolute counts of NK cells and (C) CD79a^+^ B cells in blood of alemtuzumab-treated RM (n = 8) versus human IgG-treated controls (n = 6). Results are shown as mean (+SEM) change from baseline of percentages of baseline absolute counts or percentages of Ki-67. The WRS test was used to determine the significance of differences between treatment groups (p-values ≤ 0.05 are shown).
(TIF)

S10 Fig Effect of alemtuzumab at time of ART initiation on cell-associated viral loads. (A) Comparison of SIV DNA (left panels) and SIV RNA (right panels) in PBMC and (B) peripheral LN (copies per 10^6^ cell equivalents) of alemtuzumab-treated RM (n = 8) and human IgG controls (n = 6). Threshold sensitivity varied as a function of the number of cells available for analysis; values below threshold are indicated by the gray area.
(TIF)

S11 Fig CD4^+^ memory T depletion and plasma viral load dynamics in the post-ART non-rebounders. (A) Change in absolute counts of CD4^+^ memory (T_M_) T cells (left panel) and individual plasma viral load (pvl) profiles (right panel) of alemtuzumab-treated RM (n = 6) in the pilot study. RM D6 with no post-ART rebound viremia is shown in green. (B) Change in absolute counts of CD4^+^ T_M_ (left panel) and individual pvl profiles (right panel) of RM treated with alemtuzumab at time of ART initiation (n = 8). RM G1 and G5 with no post-ART rebound viremia are shown in green.
(TIF)

S1 Data Source data for graphs used in the paper. (XLSX)
