# Supplementary figures and images for: Impact of alemtuzumab-mediated lymphocyte depletion on SIV reservoir establishment and persistence
Source: PLoS Pathog. 2024 Aug 22;20(8):e1012496. doi: 10.1371/journal.ppat.1012496 (PMC11373844; doi:10.1371/journal.ppat.1012496)

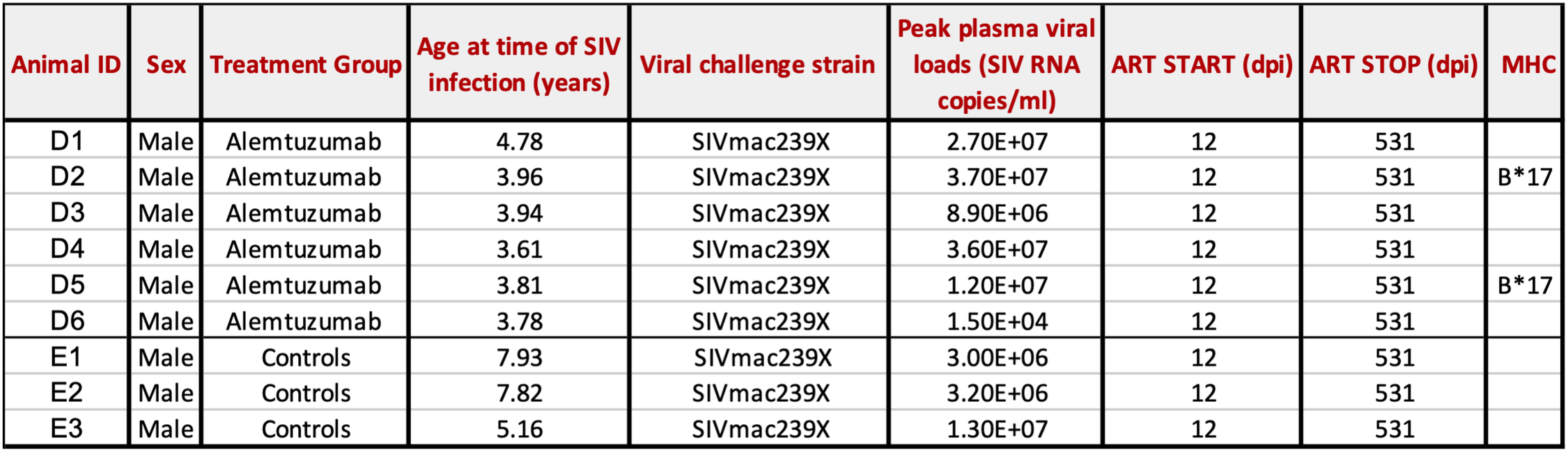

Supplement: S1 Table — The table shows the sex, age at the time of SIV infection, challenge virus, peak plasma viral loads, duration of of ART relative to SIV infection and known protective MHC-1 alleles. (TIF) [file ppat.1012496.s001.tif]

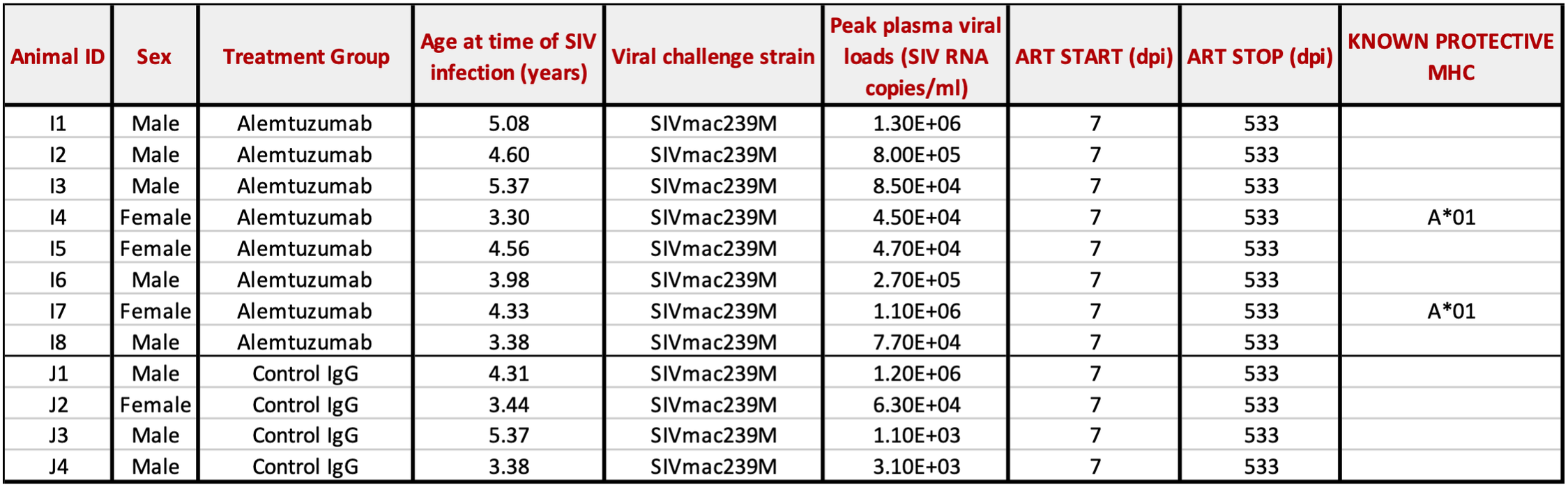

Supplement: S2 Table — The table shows the sex, age at the time of SIV infection, challenge virus, peak plasma viral loads, duration of of ART relative to SIV infection and known protective MHC-1 alleles. (TIF) [file ppat.1012496.s002.tif]

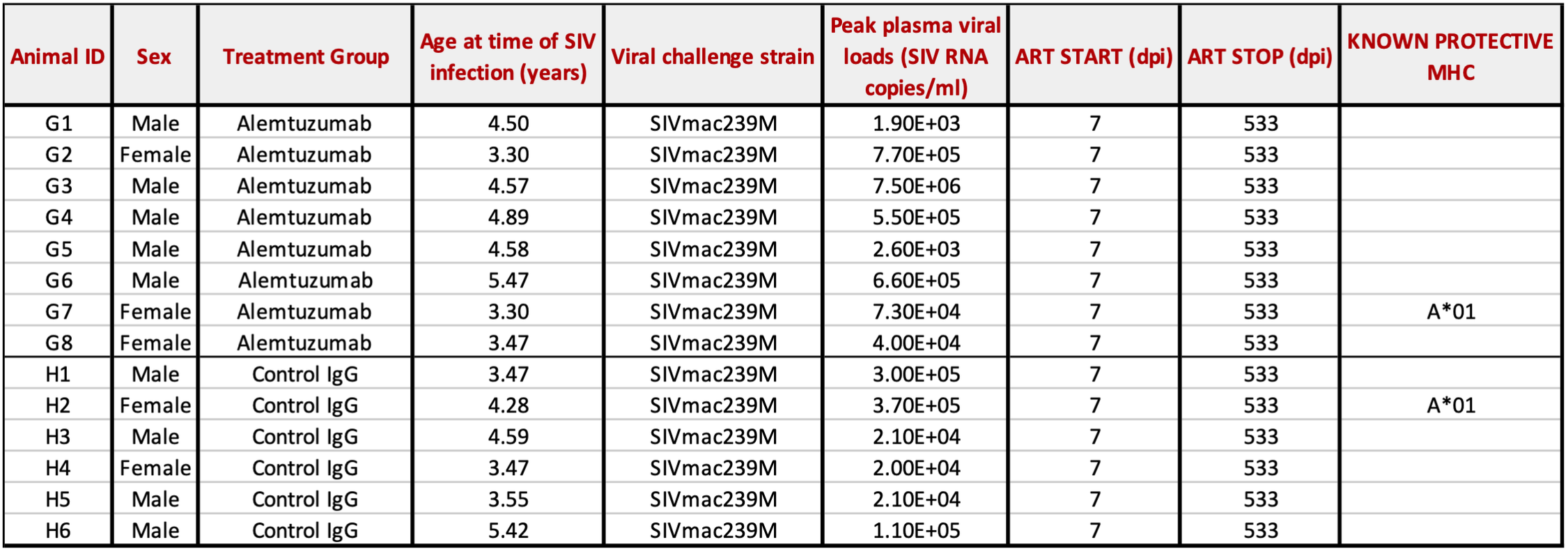

Supplement: S3 Table — The table shows the sex, age at the time of SIV infection, challenge virus, peak plasma viral loads, duration of ART relative to SIV infection and known protective MHC-1 alleles. (TIF) [file ppat.1012496.s003.tif]

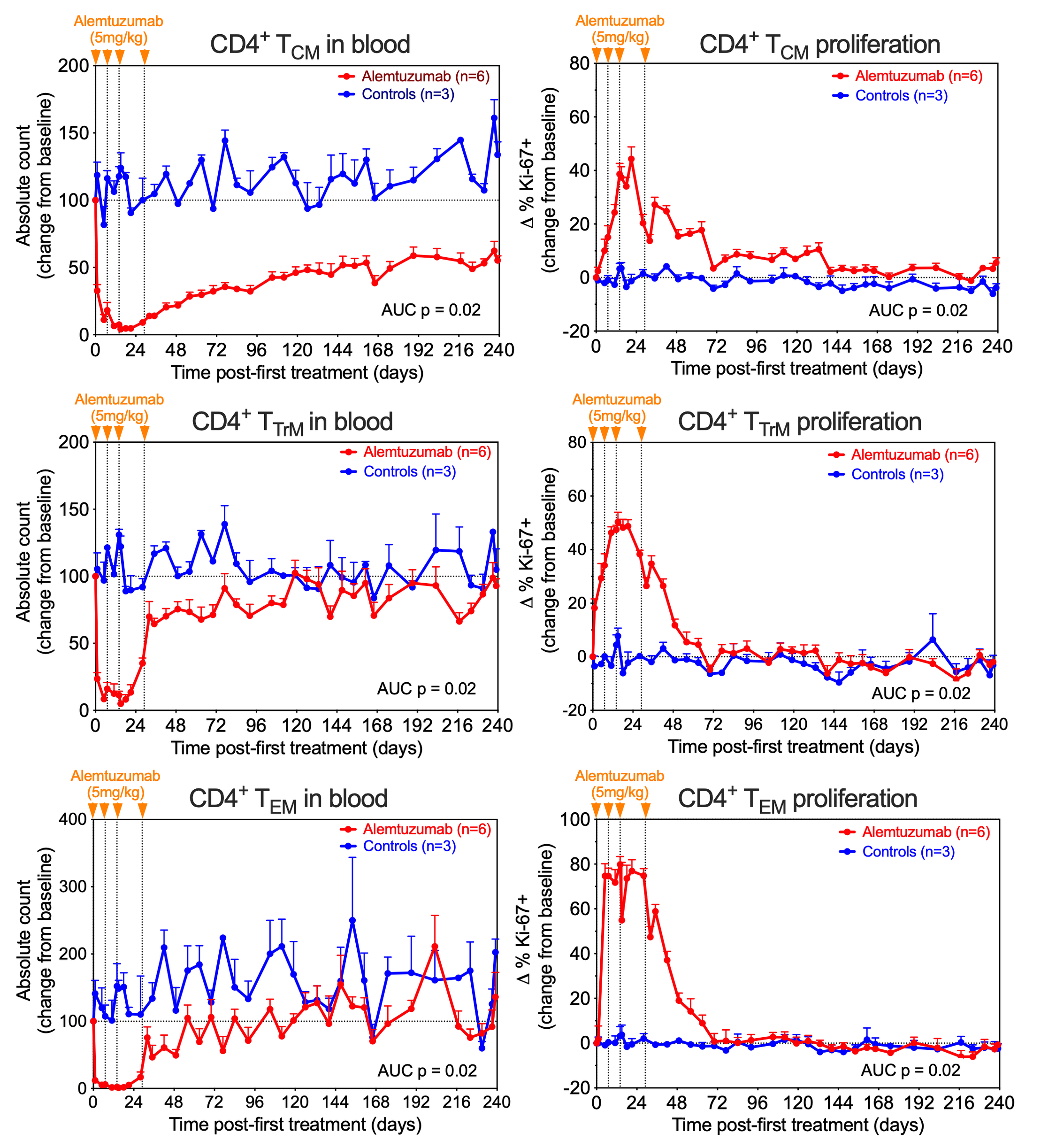

Supplement: S1 Fig — Change in absolute counts (left panels) and change in the proliferative fraction (right panels) of CD4+ central memory (TCM), transitional memory (TTrM) and effector memory (TEM) in blood following alemtuzumab (n = 6) or no treatment (n = 3). Results are shown as mean (+SEM) change from baseline of percentages of baseline absolute counts or percentages of Ki-67. The WRS test was used to determine the significance of differences between treatment groups (p-values ≤ 0.05 are shown). (TIF) [file ppat.1012496.s004.tif]

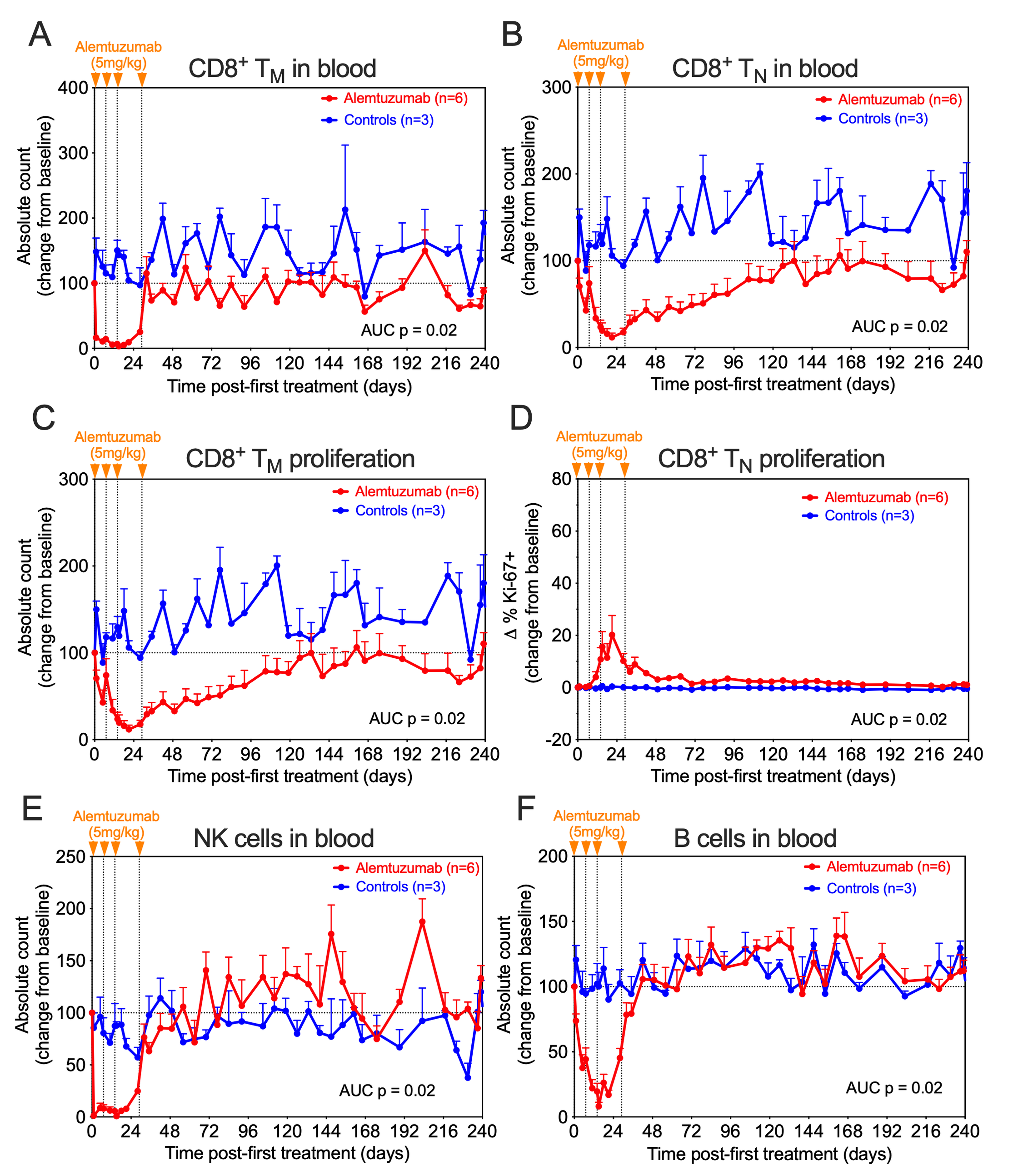

Supplement: S2 Fig — (A and B) Change in absolute counts and (C and D) change in the proliferative fraction of CD8+ memory (TM) and naïve (TN) T cells in blood following alemtuzumab (n = 6) or no treatment (n = 3). (E) Change in absolute counts of CD3- CD8+ NKG2A+ NK cells and (F) change in absolute counts of CD20+ B cells in blood of alemtuzumab-treated RM (n = 6) versus untreated controls (n = 3). Results are shown as mean (+SEM) change from baseline of percentages of baseline absolute counts or percentages of Ki-67. The WRS test was used to determine the significance of differences between treatment groups (p-values ≤ 0.05 are shown). (TIF) [file ppat.1012496.s005.tif]

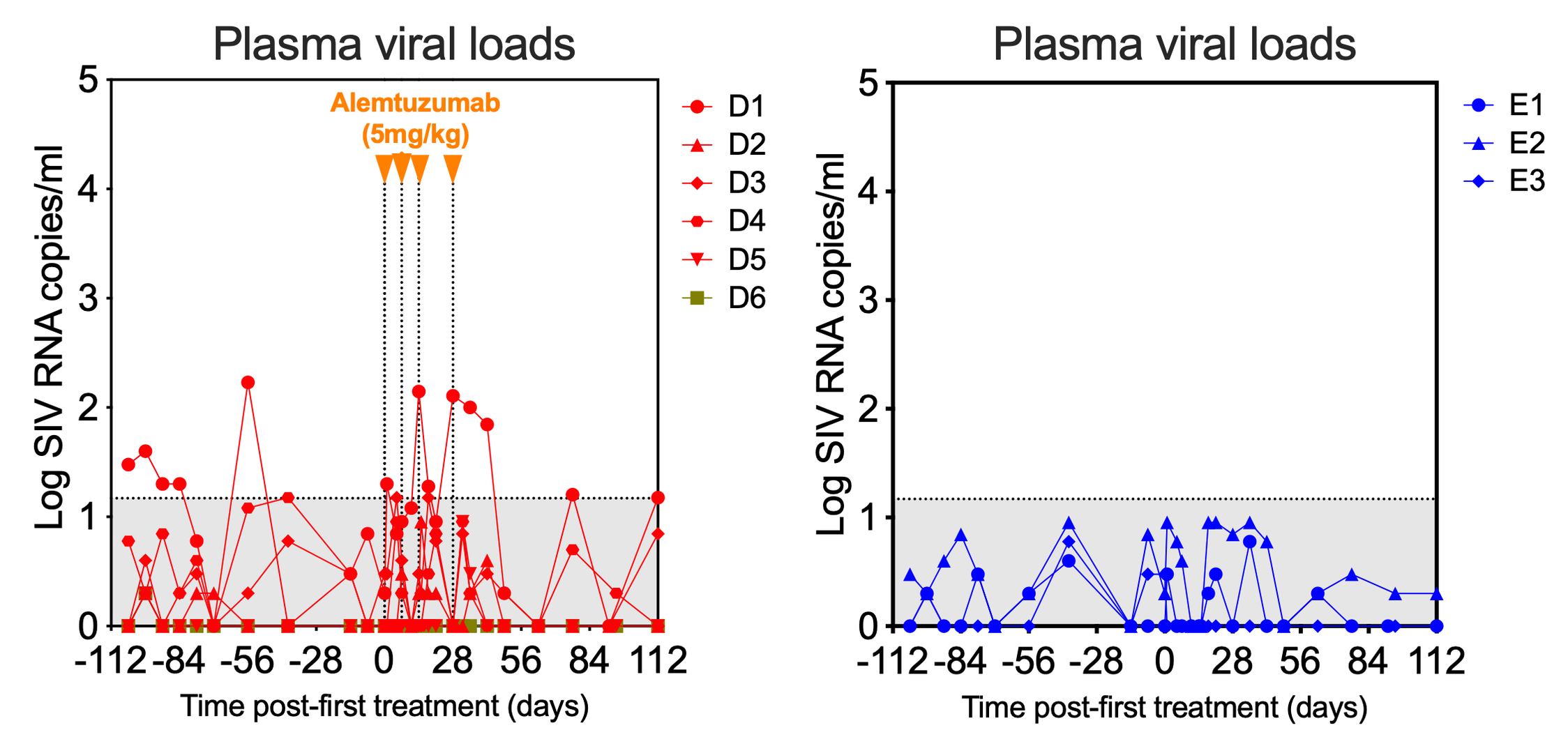

Supplement: S3 Fig — Individual plasma viral load (pvl) profiles monitored by a high-sensitivity assay (limit of detection [LOD] of 1 RNA copy/ml) prior to and during alemtuzumab treatment (n = 6) or no treatment (n = 3). The area in gray denotes pvl values below threshold of the standard assay (15 RNA copies/ml). (TIF) [file ppat.1012496.s006.tif]

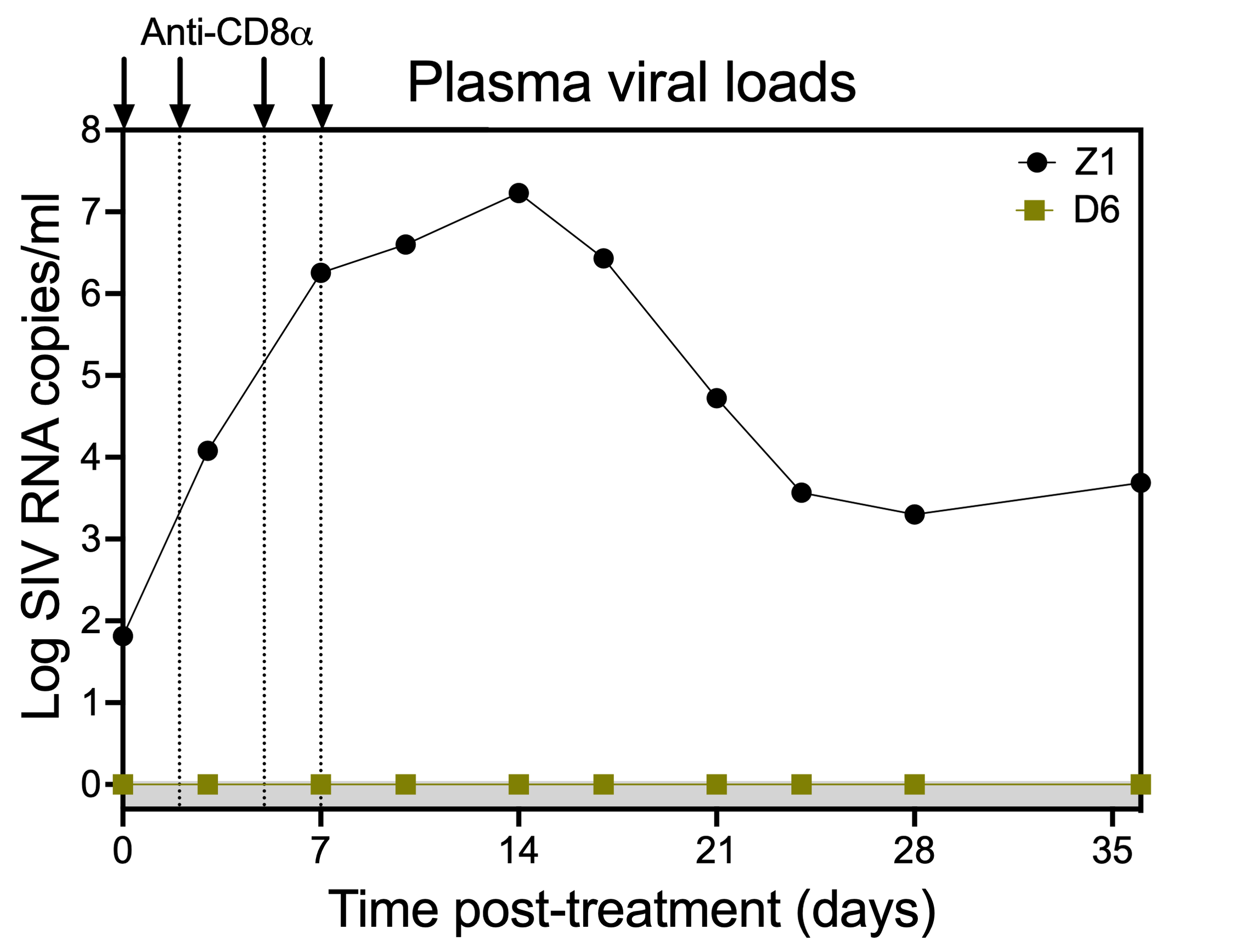

Supplement: S4 Fig — Individual plasma viral load profiles of the post-ART non-rebounder RM D6 and an SIV elite controller RM monitored by a high-sensitivity assay (limit of detection [LOD] of 1 RNA copy/ml) following treatment with the anti-CD8α depleting antibody MT807R1 at 10mg/kg SubQ on day 0 and 5mg/kg IV on days 3, 7 and 10. (TIF) [file ppat.1012496.s007.tif]

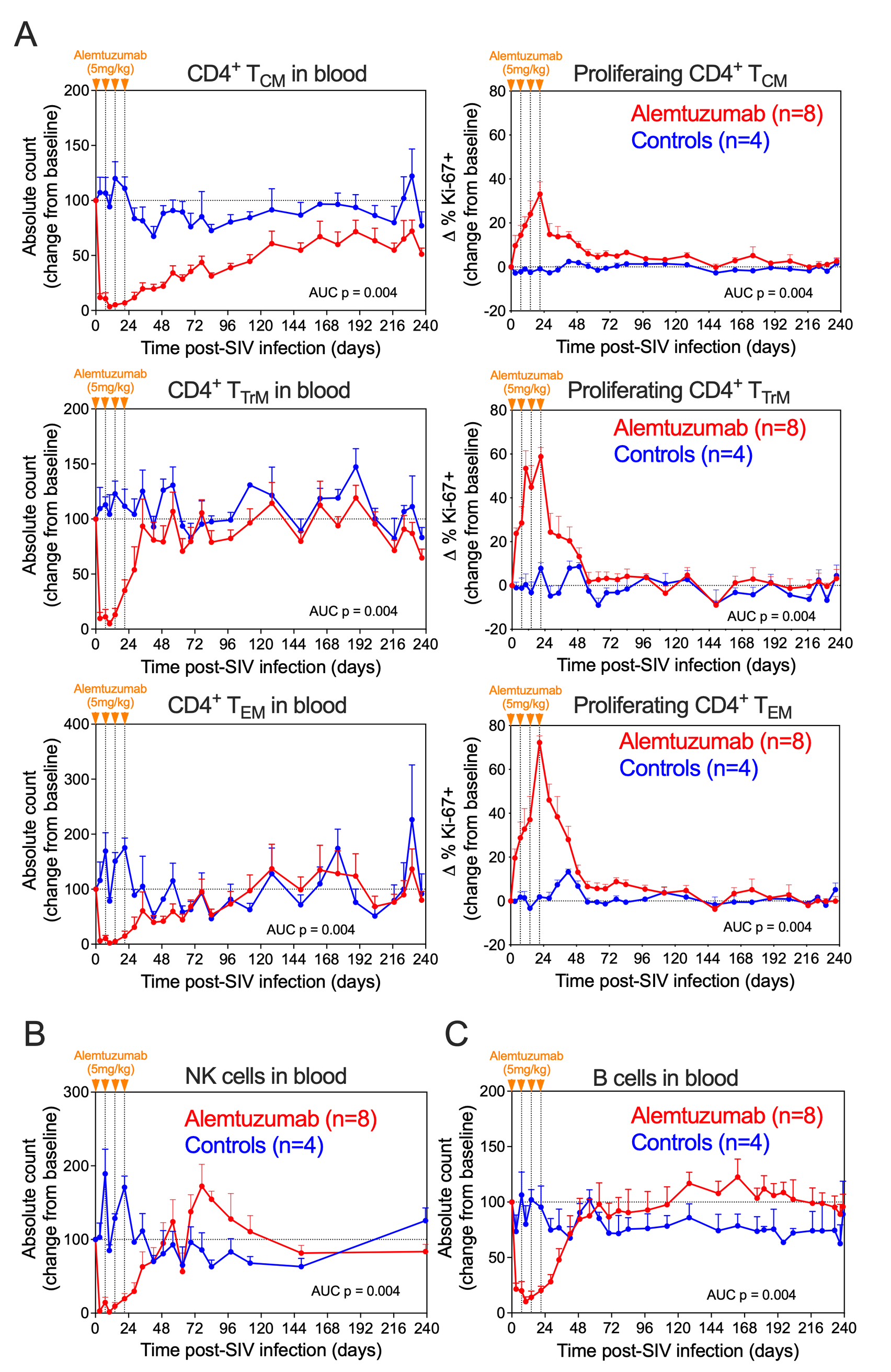

Supplement: S5 Fig — (A) Change in absolute counts (left panels) and proliferative fraction (right panels) of CD4+ memory (TM) T cell subsets, including central memory (TCM), transitional memory (TTrM) and effector memory (TEM) in blood of alemtuzumab-treated RM (n = 8) versus human IgG-treated controls (n = 4). (B) Change in absolute counts of CD3- CD8+ NKG2A+ NK cells and (C) change in absolute counts of CD79a+ B cells in blood of alemtuzumab-treated RM (n = 8) versus IgG-treated controls (n = 4). Results are shown as mean (+SEM) change from baseline of percentages of baseline absolute counts or percentages of Ki-67. The WRS test was used to determine the significance of differences between treatment groups (p-values ≤ 0.05 are shown). (TIF) [file ppat.1012496.s008.tif]

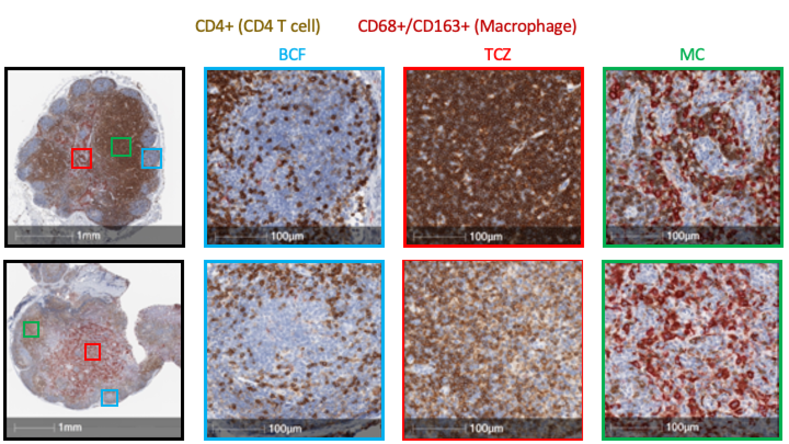

Supplement: S6 Fig — Representative images of immunohistochemical analysis performed on LN sections obtained from an RM before alemtuzumab (-26 dpi) [top] and 10 days after the last dose of alemtuzumab (41 dpi) [bottom]. Magnified images demonstrate the effect of alemtuzumab on the B cell follicles (BCF), T cell zone (TCZ), and medullary cord (MC) regions of the lymph node. CD4+ cells are in brown, while CD68+CD163+ cells are in maroon. Scale bars: 1mm-100 μm. (TIF) [file ppat.1012496.s009.tif]

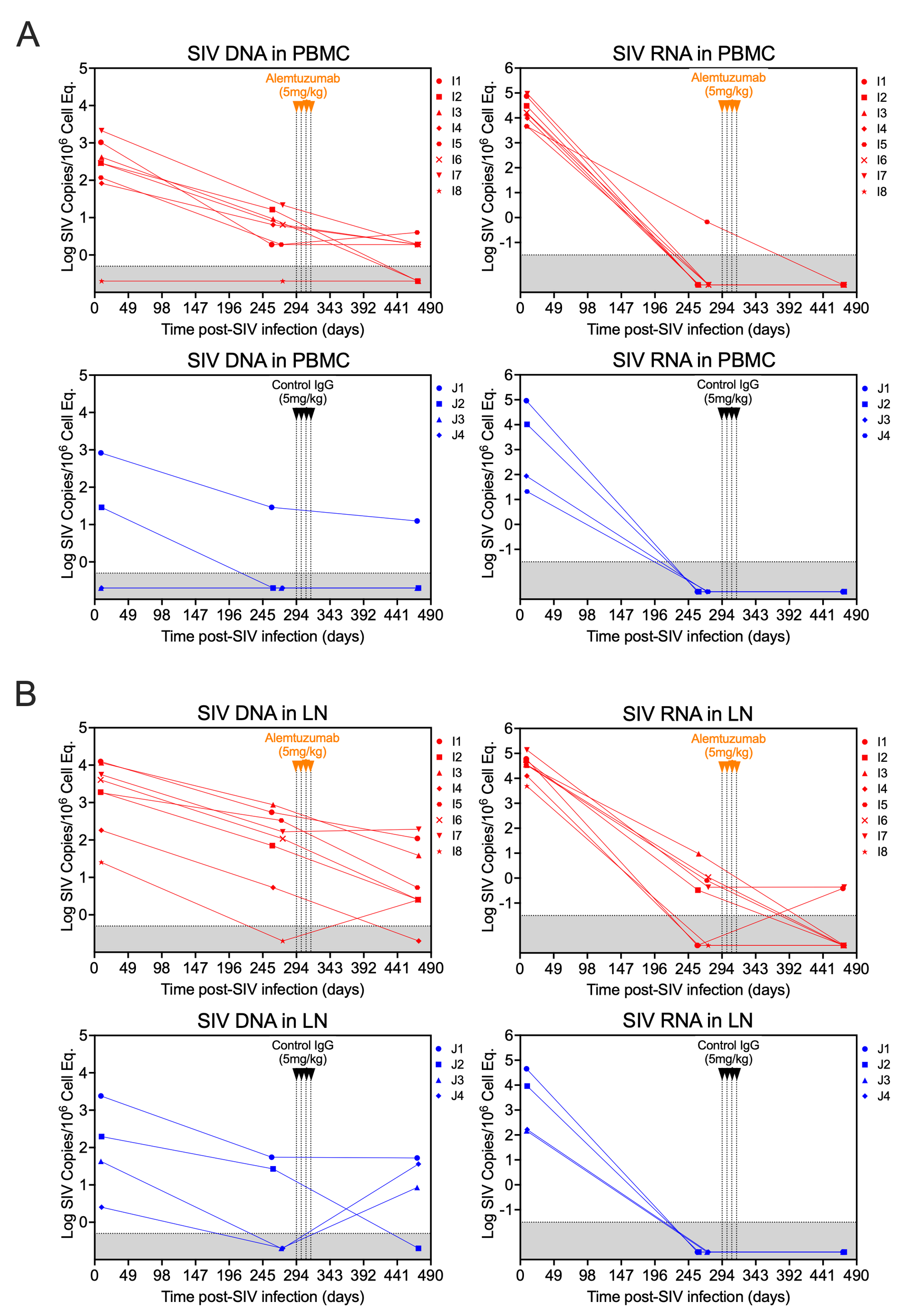

Supplement: S7 Fig — (A) Comparison of SIV DNA (left panels) and SIV RNA (right panels) in PBMC and (B) peripheral LN (copies per 106 cell equivalents) of alemtuzumab-treated RM (n = 8) and human IgG-treated controls (n = 4). Threshold sensitivity varied as a function of the number of cells available for analysis; values below threshold are indicated by the gray area. (TIF) [file ppat.1012496.s010.tif]

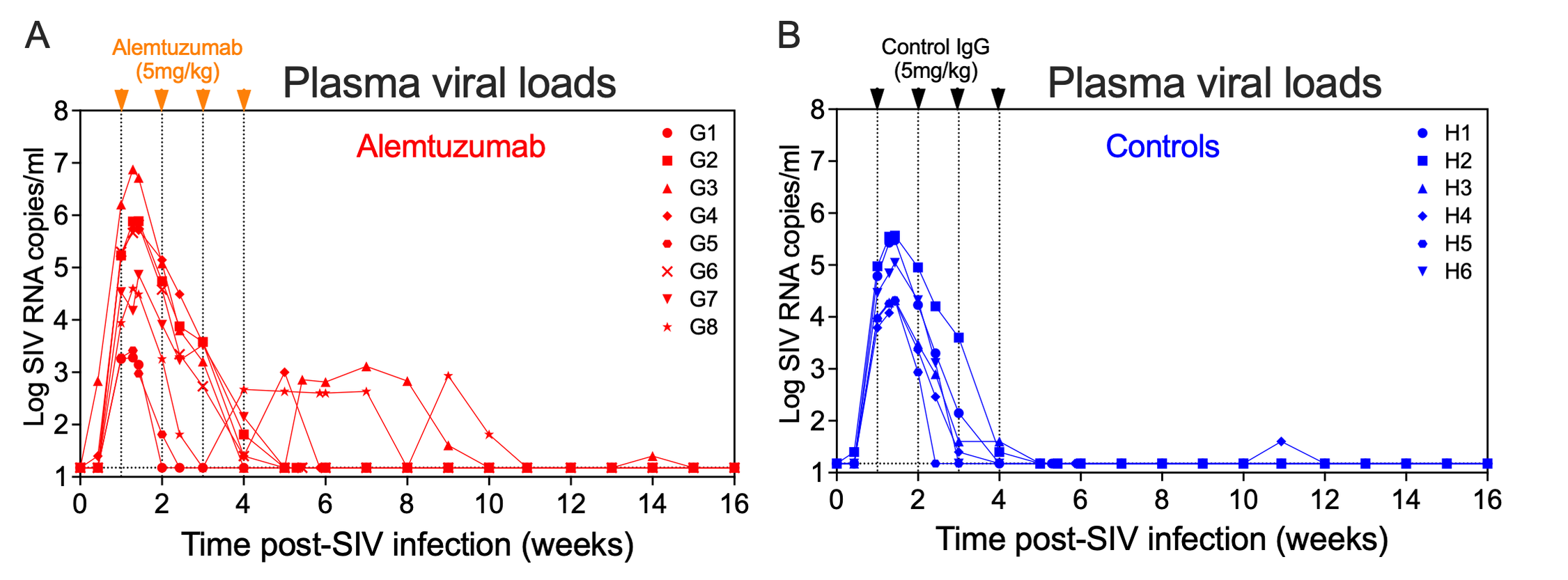

Supplement: S8 Fig — (A) Individual plasma viral load (pvl) profiles of alemtuzumab-treated RM and (B) human IgG control antibody-treated RM up to 16 weeks post-SIVmac239M infection. The dotted line indicates a pvl threshold of 15 RNA copies/ml. (TIF) [file ppat.1012496.s011.tif]

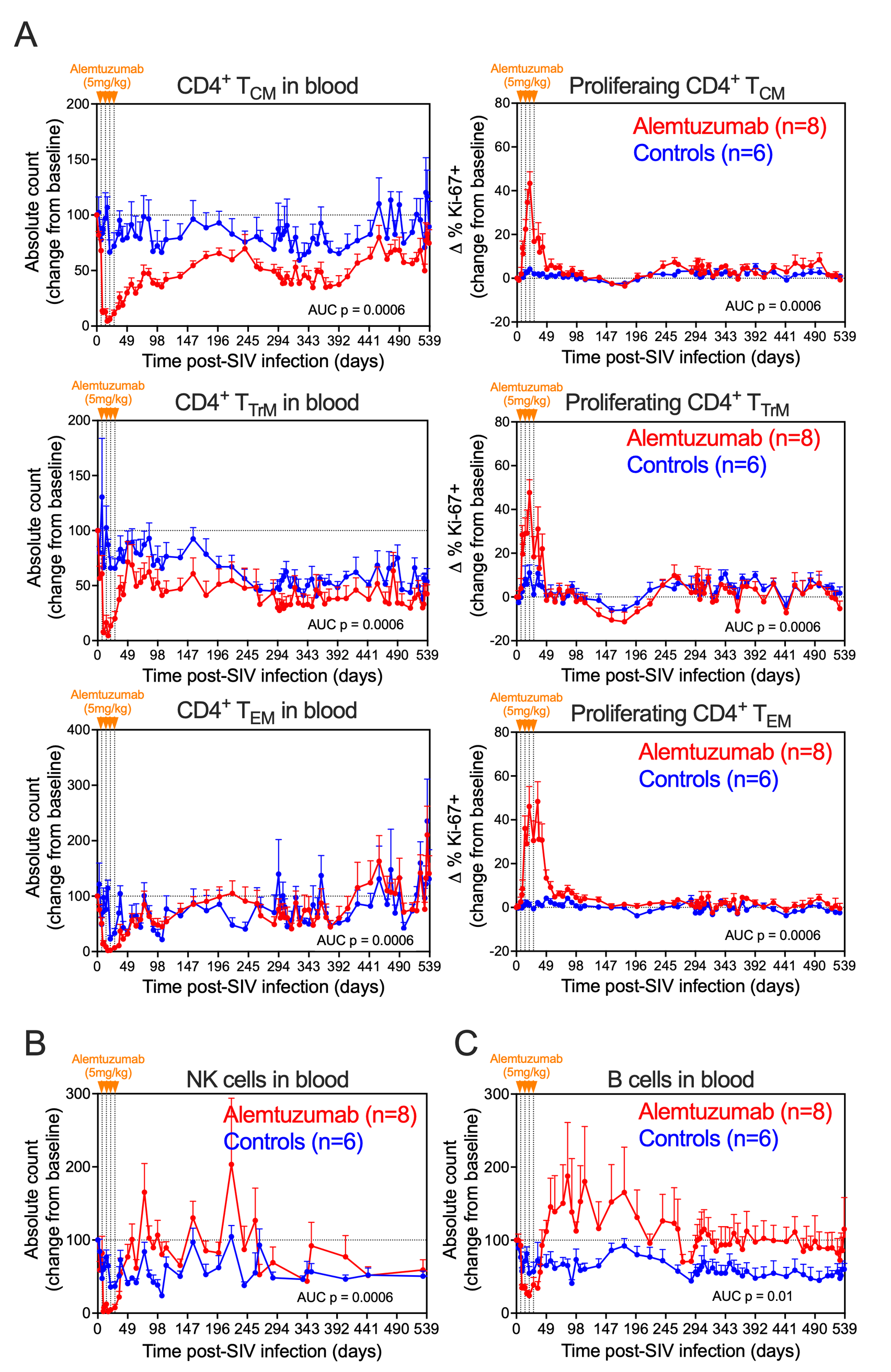

Supplement: S9 Fig — (A) Change in absolute counts (left panel) and proliferative fraction (right panel) of CD4+ memory (TM) subsets, including central memory (TCM), transitional memory (TTrM) and effector memory (TEM) in blood of alemtuzumab-treated RM (n = 8) versus human IgG-treated controls (n = 6). (B) Change in absolute counts of NK cells and (C) CD79a+ B cells in blood of alemtuzumab-treated RM (n = 8) versus human IgG-treated controls (n = 6). Results are shown as mean (+SEM) change from baseline of percentages of baseline absolute counts or percentages of Ki-67. The WRS test was used to determine the significance of differences between treatment groups (p-values ≤ 0.05 are shown). (TIF) [file ppat.1012496.s012.tif]

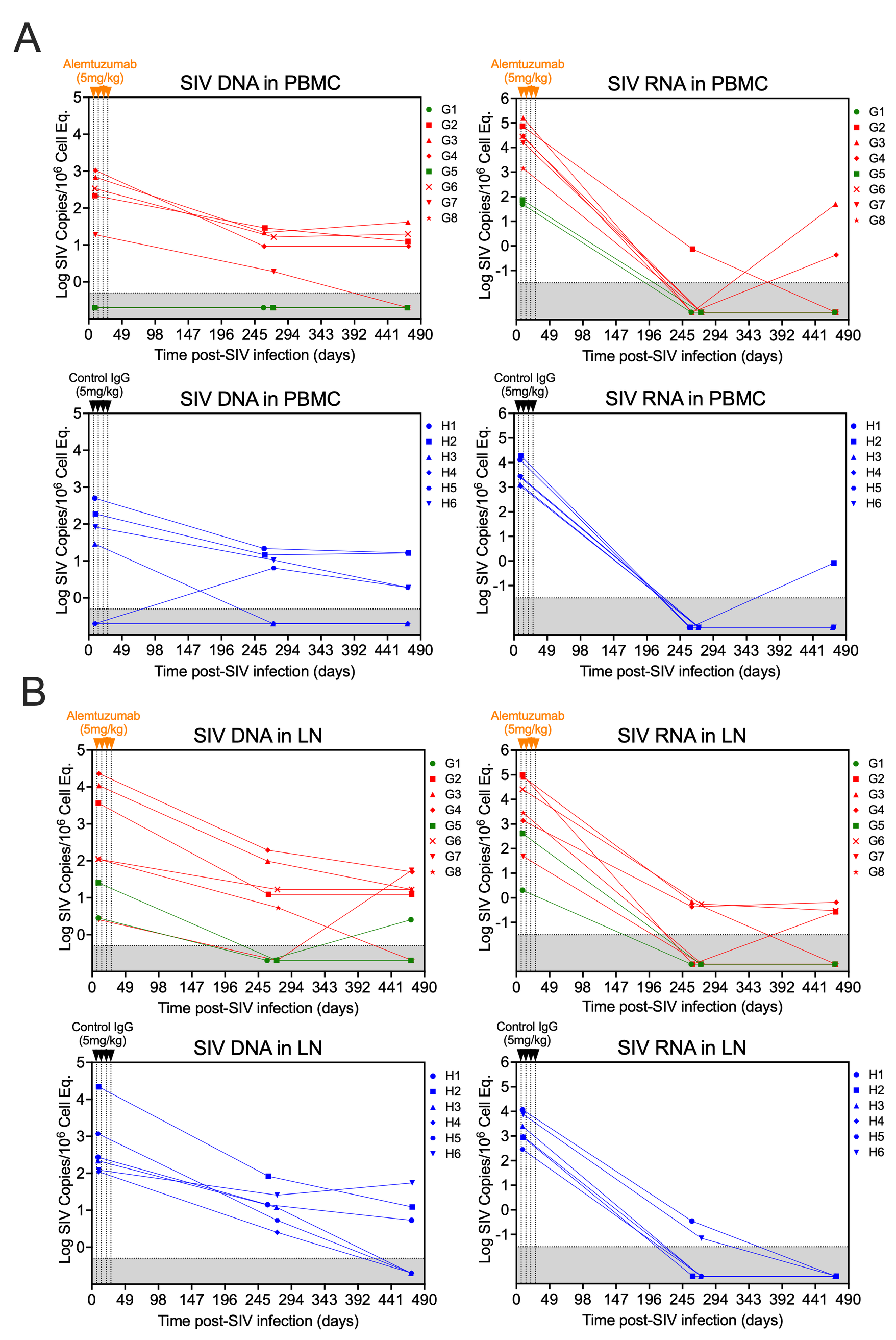

Supplement: S10 Fig — (A) Comparison of SIV DNA (left panels) and SIV RNA (right panels) in PBMC and (B) peripheral LN (copies per 106 cell equivalents) of alemtuzumab-treated RM (n = 8) and human IgG controls (n = 6). Threshold sensitivity varied as a function of the number of cells available for analysis; values below threshold are indicated by the gray area. (TIF) [file ppat.1012496.s013.tif]

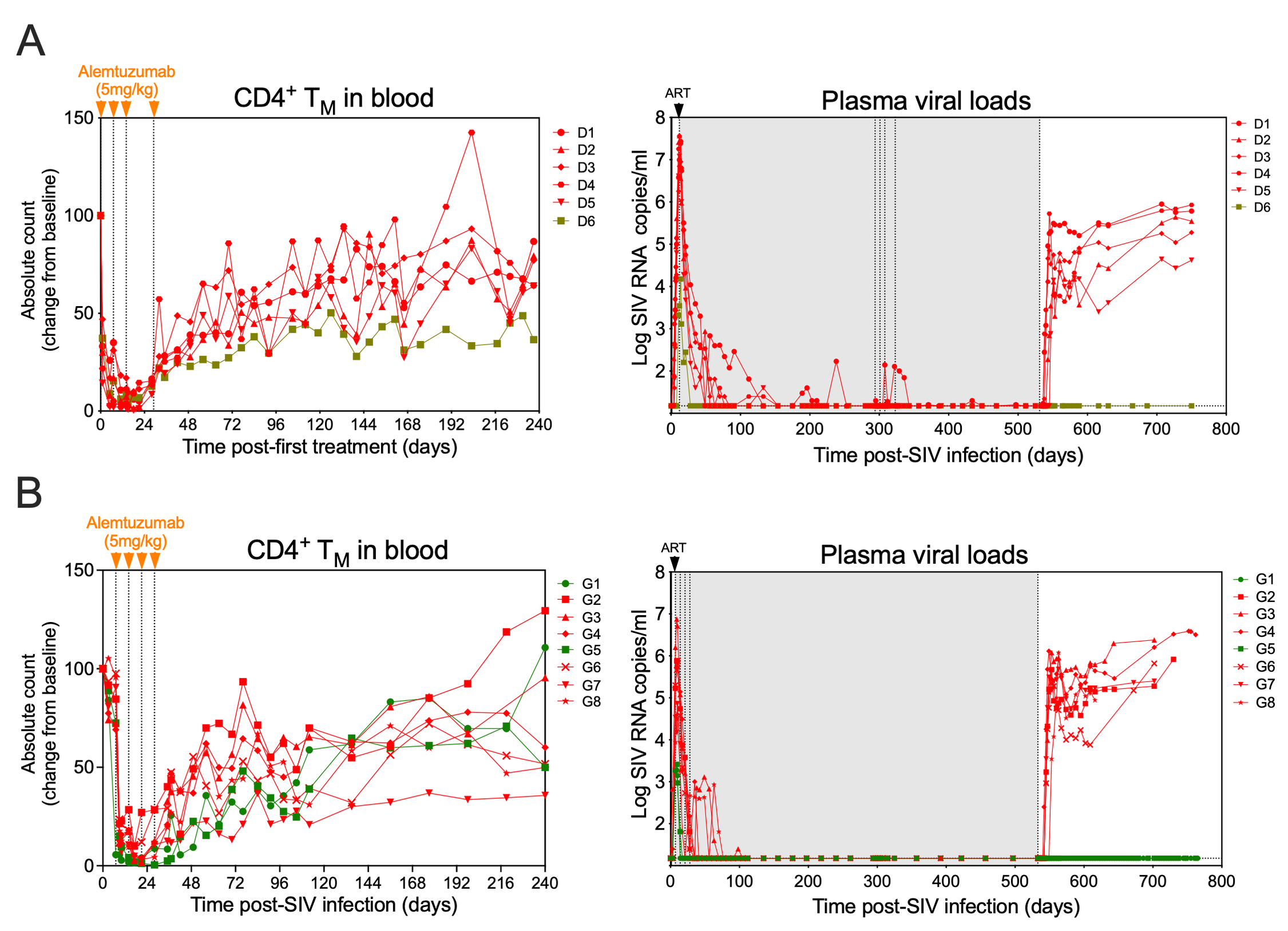

Supplement: S11 Fig — (A) Change in absolute counts of CD4+ memory (TM) T cells (left panel) and individual plasma viral load (pvl) profiles (right panel) of alemtuzumab-treated RM (n = 6) in the pilot study. RM D6 with no post-ART rebound viremia is shown in green. (B) Change in absolute counts of CD4+ TM (left panel) and individual pvl profiles (right panel) of RM treated with alemtuzumab at time of ART initiation (n = 8). RM G1 and G5 with no post-ART rebound viremia are shown in green. (TIF) [file ppat.1012496.s014.tif]
